# Synthesis, Characterization and Evaluation of Peptide Nanostructures for Biomedical Applications

**DOI:** 10.3390/molecules26154587

**Published:** 2021-07-29

**Authors:** Fanny d’Orlyé, Laura Trapiella-Alfonso, Camille Lescot, Marie Pinvidic, Bich-Thuy Doan, Anne Varenne

**Affiliations:** Chimie ParisTech PSL, CNRS 8060, Institute of Chemistry for Life and Health (i-CLeHS), 75005 Paris, France; fanny.dorlye@chimieparistech.psl.eu (F.d.); laura.trapiella@chimieparistech.psl.eu (L.T.-A.); camille.lescot@chimieparistech.psl.eu (C.L.); marie.pinvidic@chimieparistech.psl.eu (M.P.); bich-thuy.doan@chimieparistech.psl.eu (B.-T.D.)

**Keywords:** peptide synthesis, flow chemistry, peptide self-assembly, physicochemical and biological characterization, biomedical applications, nanotheranostics

## Abstract

There is a challenging need for the development of new alternative nanostructures that can allow the coupling and/or encapsulation of therapeutic/diagnostic molecules while reducing their toxicity and improving their circulation and in-vivo targeting. Among the new materials using natural building blocks, peptides have attracted significant interest because of their simple structure, relative chemical and physical stability, diversity of sequences and forms, their easy functionalization with (bio)molecules and the possibility of synthesizing them in large quantities. A number of them have the ability to self-assemble into nanotubes, -spheres, -vesicles or -rods under mild conditions, which opens up new applications in biology and nanomedicine due to their intrinsic biocompatibility and biodegradability as well as their surface chemical reactivity via amino- and carboxyl groups. In order to obtain nanostructures suitable for biomedical applications, the structure, size, shape and surface chemistry of these nanoplatforms must be optimized. These properties depend directly on the nature and sequence of the amino acids that constitute them. It is therefore essential to control the order in which the amino acids are introduced during the synthesis of short peptide chains and to evaluate their in-vitro and in-vivo physico-chemical properties before testing them for biomedical applications. This review therefore focuses on the synthesis, functionalization and characterization of peptide sequences that can self-assemble to form nanostructures. The synthesis in batch or with new continuous flow and microflow techniques will be described and compared in terms of amino acids sequence, purification processes, functionalization or encapsulation of targeting ligands, imaging probes as well as therapeutic molecules. Their chemical and biological characterization will be presented to evaluate their purity, toxicity, biocompatibility and biodistribution, and some therapeutic properties in vitro and in vivo. Finally, their main applications in the biomedical field will be presented so as to highlight their importance and advantages over classical nanostructures.

## 1. Introduction

Nanomedicine is an emerging key technology with the development of nanosystems as imaging probes or vectors of active moieties or activators. There is a challenging need for the development of new alternative nanostructures that can allow the coupling and/or encapsulation of therapeutic and/or diagnostic molecules, while reducing their toxicity and improving their circulation and in-vivo targeting. In this context, spontaneous formation of nanoarchitectures is a key issue in nanotechnologies and nanomedicine. The bottom-up strategy with coordinated interaction of building blocks (either organic or inorganic) leads to complex supramolecular assemblies [1] dedicated to various application fields such as optics, catalysis, electronics, drug delivery and molecular transport. Among natural building blocks to design smart nanomaterials, short peptides have drawn significant interest due to their simple structure, diversity of sequences and nanostructurations, relative chemical and physical stability, simplicity to be modified or decorated with biological and chemical entities, and their ability to be synthesized on a large scale [2]. Although some naturally occurring peptide self-assemblies can lead to medical disorder (amyloid fibrils), peptidic nanostructures have become an important strategy for nanomedicine due to their biocompatibility, biodegradability, robustness and their surface chemical reactivity via amino and carboxylic groups. Peptides, as short as dipeptides, contain all the molecular information needed to form well-ordered structures at the nanoscale. Peptide-based building blocks allow to control the structure and properties of well-structured nanoscale architectures, such as nanotubes, -spheres, -vesicles, -rods, -fibrils and even hydrogels, under mild conditions.

In order to obtain nanostructures suitable for biomedical applications, the structure, size, shape and surface chemistry of these nanoplatforms must be optimized and controlled. These properties depend directly on the nature and sequence of the amino acids that constitute them. Therefore, the first step for efficiently developing such nanoarchitectures is to design adequate short peptide chains and control their synthesis, i.e., the efficient amide bond formation and the order in which the amino acids are introduced during the synthesis. From the very first peptide synthesis by Theodor Curtius in 1882 [3], different methodological improvements were performed, with the solid-phase peptide synthesis, the reduction of the number of synthesis steps and process dimensions. Whatever the peptide sequences and their auto-assembly into nanoarchitectures, both entities have then to be extensively physico-chemically characterized. It is indeed crucial to control the purity and sequence of the peptides, as well as to understand the driving forces that control the peptide self-assembly, so as to design the most robust, biocompatible and biodistributable nanostructures in biological conditions. In addition to the peptide sequence, peptide auto-assembly can also be modulated or modified by external environmental factors and can lead to stimuli-responsive nanomaterials. Classical methods are employed so far (spectroscopic, microscopic and scattering methods) to determine the global self-assembled peptide nanostructures (sequence, size, diameter, charge density and shape), as well as their secondary, tertiary and quaternary structure. Due to their dynamic self-assembly processes, multiple characterization methods have to be implemented jointly, allowing for large time and length scales.

We will first present in this review a brief summary of classical synthesis methodologies and will concentrate on recent techniques such as continuous flow chemistry to gain in speed, purity and ease of production, and to promote specific self-assembly by deeply controlling the experimental conditions and coupling with adequate methods within the synthesis process. We will then present the main driving forces for self-assembly and describe the most synthesized short peptides and their identified self-assembly, i.e., peptide amphiphiles, aromatic peptides and the particular case of the diphenylalanine peptide (FF) and cyclic peptides. The interest of combining classical characterization methods for these short peptides of diverse composition will be described, so as to elucidate the interactions governing the self-assembly and the final nanostructure. The interest of analytical methods based on separation will be highlighted, as a prospective field. In the last part, the interest of these peptidic nanostructures for current or future biomedical applications will be described, going from pharmaceutical purposes, medical diagnosis and imaging, drug targeting and delivery, therapy against cancer, microbes, photo- and gene-therapy, regenerative medicine to tissue engineering.

## 2. Combination of Classical Peptide Synthesis and Assembly to Form Nanoobjects

Synthesis of peptide nanoobjects relies first on the preparation of the linear peptide by coupling amino acids, using homogenous coupling conditions, solid-phase peptide synthesis, in batch or using flow chemistry. Secondly, the peptide will auto-assemble to form the nanoobject. The auto-assembling properties of the peptide will depend on its sequence, and could be controlled by external factors like pH, temperature, organic or photo stimulation, and also by microfluidics. We will describe here some of the most recent methodologies and techniques for peptide synthesis with an emphasis on the use of flow chemistry, as well as for controlling peptide auto-assembly in a second part of this review.

### 2.1. Peptide Synthesis with Focus on Flow Chemistry

If we go back to the beginning, the first peptide synthesis was performed in 1882 by Theodor Curtius by coupling silver salt of glycine and benzoyl chloride, leading to the first N-protected dipeptide Benzoylglycylglycine (GG) [3].

Since this first milestone based on homogeneous liquid organic chemistry, lot of work has been performed, with the development of solid-phase peptide synthesis (SPPS) notably [4] to optimize and facilitate the synthesis of peptides. We will describe herein some of the recent examples.

**Scheme 1:**
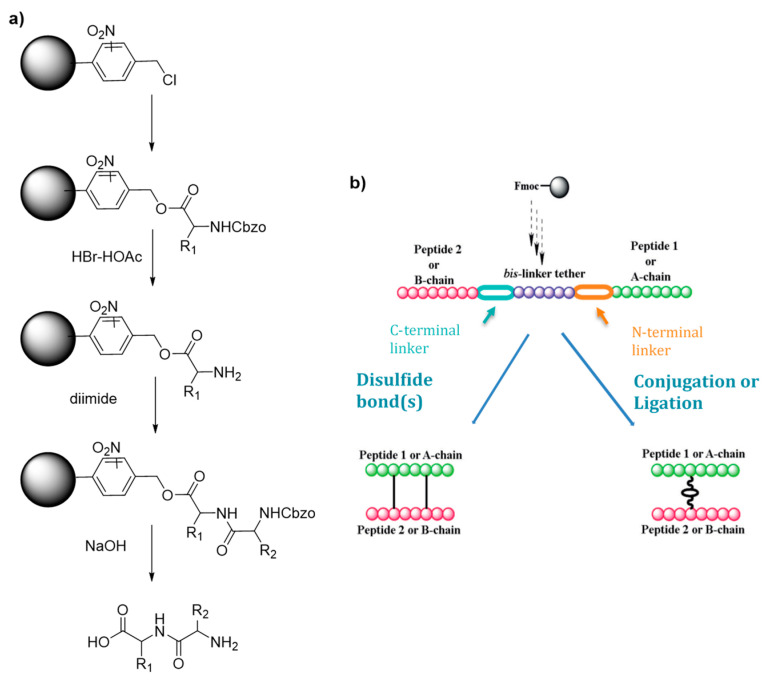
(**a**) Merrifield first solid supported peptide synthesis strategy (**b**) Hossein’s strategy for the synthesis of heterodimeric peptides. Adapte with permission from reference [9].

Merrifield [5] proposed for the first time this new approach for the synthesis of peptides relying on the stepwise addition of protected amino acids to a growing peptide chain attached to a solid resin particle through covalent bond. This strategy allows the reagents and by-products to be filtered off the resin, for a great gain of time and simplicity. In this article, the proof of concept was done with the synthesis of the model tetrapeptide l-leucyl-l-alanylglycyl-l-valine (LAGV) Scheme 1a.

Beyond linear peptides, cyclic and heterodimeric peptides are very attractive to synthesize because of their very interesting biological properties for various applications [6]. It is known that Cyclic Peptides (CP) can auto-assemble to form peptide nanotubes, thanks to the backbone-backbone stacking due to hydrogen bonding between antiparallel β-sheet oriented from the N-H bond of one CP to a C=O of another CP. In 2016 our team performed the design, synthesis and characterization of new cyclic D, l-α-alternate amino acid peptides [7]. As the formation of CP nanotubes depends on their sequence, three series of novel cyclic peptides were synthesized: each series was varied in chain length and amino acid nature, leading to 8 CPs of different van der Waals inner diameter and different properties for future applications. The synthesis of the linear peptide sequence (first step) was undergone by classical SPPS and orthogonal protection methods, followed by the cyclization step using propane phosphonic acid. While the linear peptide was obtained in a relatively good purity, the cyclization was performed in 40–90% yield, higher than the previously reported methodologies [8].

Although their synthesis is described as complicated and low yielding, heterodimeric peptides which are cystine-rich represent also highly interesting drug targets. Hossain and his group worked on an improved synthetic route to heterodimeric peptides, which reduces the number of synthesis steps compared to classical methodologies [9]. Usually, the two chains are synthesized separately, released from the solid support and submitted to multistep solution-phase reactions to control the disulfide pairing. Hossein’s protocol consists in the sequential synthesis of both chains on the same solid support separated by a chemically cleavable bis-linker (Scheme 1b). The linear dipeptide linked by the tether is then released and used for the formation of the intra peptide-peptide compound by disulfide or thioether bond. The tether is finally cleaved by a 5% hydrazine buffer, leading to the desired conjugate with an overall yield of 27%. In a simpler way, Hossain and colleagues also developed an improved SPPS synthesis strategy using orthogonally protected monomeric building blocks, which they successfully applied to the synthesis of insulin by incorporating the thioether moiety in place of the A6–A11 cystine bridge [10].

Although the SPPS has revolutionized peptides synthesis, a review underlined in 2009 the need for more powerful methods for the synthesis of peptides with longer chains [11]. The SPPS is and will be anyway part of the current developments of new methodologies for peptide synthesis. Among those, we have chosen to concentrate our attention on methodologies relying on continuous flow chemistry. Before addressing the question of solid or liquid phase process, we might look back at the center of peptide synthesis: the amide bond formation. The efficiency of this chemical reaction is determinant for the whole process. Amide bond formation relies on the activation of the carboxylic acid part, followed by the coupling with the amine part of another amino acid. A large panel of coupling reagents is available for the chemists, and the question has been well documented [12]. Takahashi and co-workers developed in 2014 a highly efficient amide bond formation methodology, relying on the very rapid activation of carboxylic acids before their also rapid conversion into amides [13]. They achieved in a microflow synthesis reactor an activation time of 0.5 s, with a reaction time of 4.3 s, allowing the synthesis of peptides with excellent yields, the lowest being 74%.

**Figure 1:**
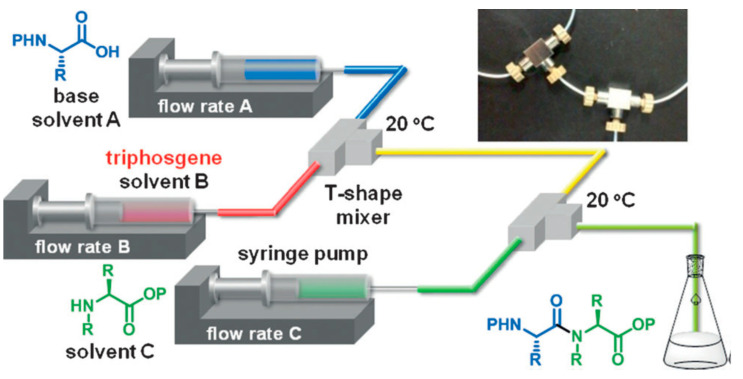
Microflow synthesis of peptides based on rapid and strong activation of carboxylic acids. Adapted with permission from Reference [13].

This methodology is based on the concept of “flash chemistry” i.e., the quasi instantaneous activation and reaction of chemical compounds, reducing the formation of by-products, and, in the case of peptides, epimerization. This is one of the first examples of the use of flash chemistry for amide bond formation. The microreactor consisted in the combination of 2 T-shape mixers (Figure 1), the first one dedicated to the activation of the carboxylic acid part of the first amino acid (flow rate A on Figure 1), with triphosgene (flow rate B on Figure 1), and the second to the coupling with the protected amino acid (flow rate C on Figure 1). The whole microsystem was connected by Teflon tubing and flowed by syringe pumps. This process afforded the coupling of 2 amino acids in a very short time (less than 5 s residence time) in very good yield and purity. The authors compared their flow system with the batch equivalent and showed highly superior results in flow in every case (97% yield in flow vs. 74% in batch for the best results). This study illustrates the high potential of flow chemistry for peptide synthesis.

In parallel, we can underline the work of Pentelute and his team on the development of a rapid flow-based peptide synthesis methodology [14] and its successful concomitant application to the total synthesis of DARPin pE59 (Designed Ankyrin Repeat Protein, 130 amino acids residues) and Barnase (RNase *B. a.*, 113 amino acids) [15]. Their methodology consisted in a fully automated solid-phase peptide synthesis in continuous flow, allowing the incorporation of one amino acid every 1.8 min. The main advantage of this work compared to the previous literature is the design of a low-volume and low-pressure reaction vessel. This could overcome the problems of high volume of wash solvent, and of low flow rates required due to high pressures of the systems. This vessel allowed the authors to impulse high flow rates, so that the reaction time is shorter, without raising the pressure too high. This methodology allowed the authors to synthesize native DARP in pE59 and Barnase proteins through the rapid and efficient production of high-quality peptide fragments which were assembled via convergent N, C Ligation. The final full lengths proteins (130 and 113 residues) were found to be biologically active. Three years later the same authors improved their protocol, providing a SPPS approach where the amide bond formation takes only 7 s and the total synthesis time is 40 s per amino acids [16].

Among the recent developments presented in recent reviews [17,18], the group of Seeberger in 2019 improved the methodology to overcome some limitations of solid support reactors [19]. Indeed, as main continuous flow protocols are developed with fixed bed reactors, the two major drawbacks are the reagent channeling and the high back pressure. Therefore, Seeberger and his team envisaged the development of a variable bed reactor. A differencial pressure sensing was applied to monitor pressure changes across the reaction bed caused by resin swelling and shrinking, leading to autonomous adjustments made by a piston to the resin bed size for maintaining the pressure while the resin swells freely.

Compared to a fixed bed reactor where the reactions are monitored in line, a variable bed allows a real time monitoring of the elongation efficiency and peptide tertiary structure, while maintaining a low overall system pressure throughout peptide syntheses. This new reactor could also enable tracking of problematic couplings, on-resin aggregation, and further understanding the effects of some synthetic conditions on peptide sequences.

Generally, peptide synthesis gains speed and synthesis control via continuous flow methodologies. Gain in the reaction time is very important in the field of peptide synthesis for nanostructures, since the assembly of the peptide relies mainly on the amino acid sequence. The use of continuous flow would thus allow several fast assays and changes in the amino acids sequence to have in hands a panel of peptidic structures for studying their assembly properties.

### 2.2. Self-Assembly to Form Peptide Nanoobjects

The assembly of nanopeptides is influenced by the conditions of the solution, such as pH, temperature, ionic strength, salt or solvent nature. We will present here some articles highlighting the influence of these three parameters of the environment on the self-assembly of peptides to form nanostructures, as well as some of the techniques developed to promote this assembly; the intrinsic properties related to the sequence itself will be further explored in the next session of this review.

Some literature highlights the influence of pH on nanostructuration. For example, the KLVFFAE peptide sequence of Alzheimer’s disease (AD) was demonstrated to be very sensitive to environmental pH as it was shown to auto-assemble into fibers at neutral pH and into tubes at acidic pH [20]. Ghosh et al. also developed a strategy for precisely controlling the self-assembly of the Peptide Amphiphiles (PAs) by adjusting the pH of the solution [21]. They found that PAs could self-assemble into nanofibers at pH 4 and spherical nanomicelles at pH 10.

Another illustrative example has been published by the group of Fojan [22] in 2010. The authors present the synthesis and characterization of a novel amphiphilic peptide KA6 which exhibits a clear charge separation controllable by the pH of the environment. As the self-assembly of this system is largely governed by electrostatic interactions, a modification of the pH causes a modification of the micellar structure, revealed by atomic force microscopy (AFM) and circular dichroism (CD) characterizations (see part III). At basic pH, the micellar structure is inverted, exposing the opposite end of the peptide chain to the solution, going from pH 2 to pH 11.

l-Carnosine (β-alanine-histidine, βAH), is a peptide providing a large range of biological activities. Peptide βAH is highly water-soluble, but it does not self-assemble in water. Castelletto and Hamley explored the construction of novel βAH supramolecular self-assemblies [23]. Their strategy to drive βAH self-assembly involves turning the dipeptide into a PA through the lipidation of βAH by adding a C16 palmitoyl lipid chain to the peptide by classical homogeneous synthesis techniques. They further demonstrated that a peptide amphiphile undergoes reversible thermal transition between nanotubes and helical ribbons and twisted bands at higher temperature [24]. The nature of this transition was elucidated using a combination of microscopy, x-ray scattering and spectroscopic methods (see part III). This transition implies a change of curvature of the PA bilayer, which can be due to changes in the solubility of the peptide caused by temperature changes in hydrogen bonding, both in the β-peptide sheets and with the water solvent molecules. In the context of their study of the amyloid-like nanosheet peptide (KLVFFAK) as a retrovirus carrier, Liu and coworkers found that the size and yield of amyloid type nanoscale foil can be fine-tuned by changing the ionic strength in aqueous solution [25]. While increasing the concentration of NaCl (from 0 to 1 M), the width of the nanoparticle keeps increasing from 0.2–0.4 μm to 0.6–1.0 μm with a plateau at 0.5 M, and the yield (% of peptide in solution) increased also with the NaCl concentration. A similar trend was observed in the presence of MgCl_2_, but the plateau appeared at a lower concentration due to the strongest ionic contribution of the divalent magnesium cation. The authors suggest that the salt addition may improve the aggregation capacity by eliminating repulsive interactions between positively charged KK contacts, which are concentrated on the surfaces of the nanoparticle. In addition, it has been observed that the morphology of the nanoparticle is stable for more than 20 days at 37 °C under stirring, indicating that it is thermodynamically favorable.

Acuna and Toledo studied both the self-assembly of the diphenylalanine peptide (l-Phe-l-Phe, FF) dissolved in water and the effect of electrolyte type, concentration and pH on the formed nanostructures. [26] SEM and TEM were used for characterization of the different structures obtained (see part III). Results show that FF nanotube formation through self-assembly is a fine balance between electrostatic, hydrogen bonding, and hydrophobic interactions; any perturbation in these equilibria can prevent nanotube formation. Salts, such as NaCl and CaCl_2_ (at 50, to 200 mM concentration), have been found to promote the formation of very long nanotube structures. This would be due to a screening effect and the fact that cations are layout-forming and stimulate hydrophobic interactions; therefore, nanotube assembly occurs and also benefits electrostatic interactions, hydrogen bonds, and longer nanotubes. The presence of AlCl_3_ produces an imbalance in the electrostatic interactions and hydrogen bonding because of excess Cl^−^, a structure-breaking anion that impedes the nanostructure formation.

**Figure 2:**
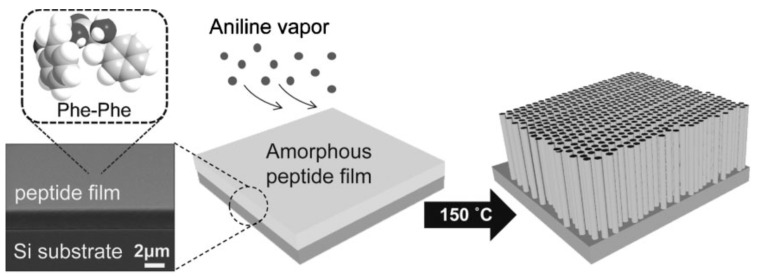
Peptidic nanowires vertically grown under aniline vapors. With permission from Reference [27].

Besides the control of the external environmental parameters influencing peptide auto-assembly, we can underline some synthesis protocols and methodological tools which can be used to drive on-line self-assembly of peptides. The group of Park in 2008 reported a novel solid-phase growth of crystalline peptide nanowires at high temperatures driven by aniline vapor under anhydrous conditions [27]. For this study, an amorphous peptide thin film was prepared by drying a drop of 1,1,1,3,3,3-hexafluoro-2-propanol (HFIP) solution containing diphenylalanine on a Silica substrate. Since water vapor could modify the surface structure of the peptidic thin film the experiments were conducted under anhydrous conditions (vacuum dessicator). From the amorphous peptide film, the authors were able to grow vertically well-aligned peptide nanowires by aging the film at temperatures above 100 °C with aniline vapor (Figure 2). The specific influence of the aniline vapor was studied by aging the film at different temperatures, with or without aniline vapor.

At 50 °C, no change in the film was observed in the absence of aniline, whereas thick nanorods were formed in the presence of aniline. At temperatures of 100 and 150 °C, and with or without aniline vapour, one-dimensional nanostructures were formed, but with different shapes: while the high-temperature aniline vapor aging resulted in the formation of uniform and well-aligned peptide nanowires, dry air aging without aniline at the high temperatures promoted the growth of highly flexible nanofibrils with an irregular shape, illustrating the crucial role of aniline vapors in this study. According to the authors, this role relies on the presence of the amine part of aniline, which can be a hydrogen-bond donor. This hypothesis might require further experimentation to be validated, but it seems plausible since toluene and benzene vapors did not show any change due to the amorphous FF film.

More recently, Yan and co-workers explored the role of trace solvent in the dipeptide self-assembly [28]. In this work, they discovered that a trace amount of solvent may be a dominant factor for directing and mediating self-assembly of FF. The FF/dichloromethane (CH_2_Cl_2_) solution (from Commercial FF) was selected as a model, and compared to three other types of solvents. Type I solvents (such as ethanol, DMF and acetone) have hydrogen-bonding interactions with FF. Type II solvents (toluene) can lead to possible π-π interactions with FF. Type III solvents (n-hexane) can generate van der Waals interactions with FF. The optical microscopy images of samples in solution showed that FF underwent crystallization in pure CH_2_Cl_2_, whereas gelling occurred when a trace amount of hydrogen-bond-forming solvent (type I) was added in CH_2_Cl_2_. Therefore, in pure CH_2_Cl_2_, crystallization was favored with the growth of FF into each dimension at a comparable rate. When hydrogen-bond-forming solvents were added to CH_2_Cl_2_, directional hydrogen bonding would drive the assembly of FF molecules in one dimension and resulted in the formation of fibers (in ethanol) or even ribbon structures (DMF or acetone). On the contrary, the addition of a trace amount of toluene and n-hexane did not promote the formation of fiber structures. These results highlight the key role of hydrogen bonding in the formation of fibers, which can be tuned by controlling solvent composition.

**Scheme 2:**
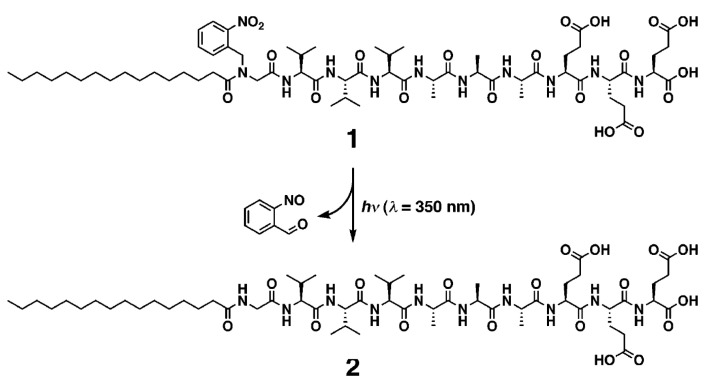
Photochemical cleavage of 2-nitrobenzoyl group in PA 1. Reprinted with permission from Reference [29].

Some articles present synthetic processes to promote specific self-assembly, with the help of photochemistry light-impulsion or enzymatic stimulators. Inspired by the switchable structures in biomolecules, Stupp and his group have investigated the synthesis of photoresponsive PAs, well-known to self-assemble into supramolecular nanofiber [29]. They reported the discovery of a quadruple helical fiber formed by photoresponsive PA 1 (Palmitoyl tail-nitrobenzoyl group-GV3A3E3.) and its conversion into single fibers upon photochemical cleavage of the 2-nitrobenzyl group in 1 (Scheme 2).

The amphiphilic structure of PA1 is expected to promote self-assembly into cylindrical nanofibers. The nitrogen of the N-terminal amide of PA1 has a 2-nitrobenzyl group that can be cleaved by irradiation at 350 nm to afford PA2. The lack of hydrogen bonding on the amide closest to the alkyl chain and the bulkiness of the 2-nitrobenzyl group made the authors expecting that PA1 and PA2 would differ in their supramolecular architecture after self-assembly. A transmission electron microscopy (TEM) image of one of the supramolecular structures revealed a quadruple helix, which was previously very scarcely described. Interestingly, after a 5-min irradiation of PA1, the helical structures disappeared completely in the TEM images, and only cylindrical fibrils with a diameter of 11 nm were observed. In matrix-assisted laser desorption ionization-time of flight-mass spectrometry (MALDI-TOFMS) spectrometry, the signals corresponding to PA2 were clearly observed after photoirradiation and high performance liquid chromatography (HPLC) showed nearly complete conversion from PA1 to PA2.

**Scheme 3:**
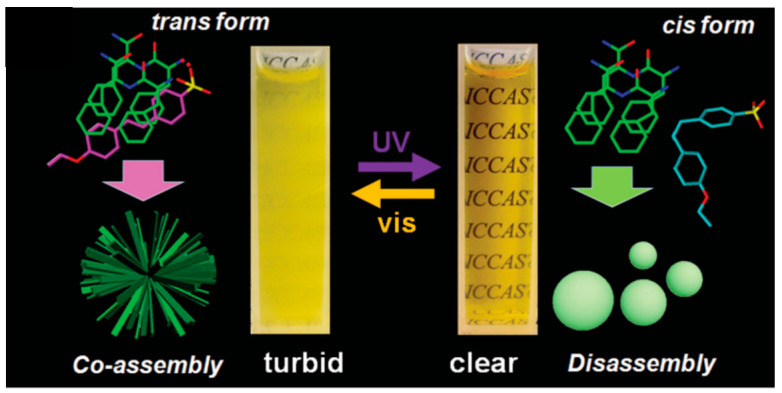
Photoswitchable system for auto-assembly of CDP. Reprinted with permission from Reference [30].

Light-impulse assembling has attracted considerable attention because it is reversible, fast, and works remotely without generating any undesired substances. In a report from 2015, Li designed a photoswitchable sulfonicazobenzene 4-[(4-ethoxy)phenylazo] benzenesulfonic acid (EPABS), with the aim of optically manipulate the self-assembly of a cationic FF peptide (CDP, H-Phe-Phe-NH_2_·HCl) [30]. The photo-induced trans–cis conformational change of EPABS significantly influenced the peptide assembly and a reversible structural transition between a branched microstructure and a vesicle-like nanostructure was observed (Scheme 3). Both SEM and TEM images indicated that branched structures were generated through co-assembly of CDP and EPABS. The detailed SEM images revealed that the branched nanostructures are built by elongated nanoplates and helical nanobelts that are interconnected through a center core to build the final co-assembled structure

The authors proposed a possible mechanism of the assembly and transformation process. Before ultra-violet (UV) illumination, trans-EPABS is shown to be inserted into the CDP molecular arrangement thanks to electrostatic and π-π interactions. According to Fourier-Transform Infra Red (FTIR) measurements, the aromatic rings of EPABS overlap with the CDP aromatic rings, leading to the formation of branched structures (on the left on Scheme 3). After being irradiated by UV light, trans-EPABS in the branched structure is gradually transformed into cis-EPABS. The higher hydrophilic property and steric hindrance of cis-EPABS may conduct its leaving from the branched structures, signaling a disassembly of the structure. Free CDP molecules undertake a self-assembly procedure to form vesicle-like edifices. When this system is then exposed to visible light, EPABS would return to its trans-form, leading to the previous co-assembled branched structures. Other external factors have been demonstrated to promote peptide self-assemblies, like enzymatic stimulators [31] but we have chosen not to detail those in this review.

Finally, new technologies were developed to control self-assembly of peptides into nanoobjects. In 2016, the groups of Knowles and Gazit developed a microfluidic technology for controlling peptide self-assembly [32].

**Figure 3:**
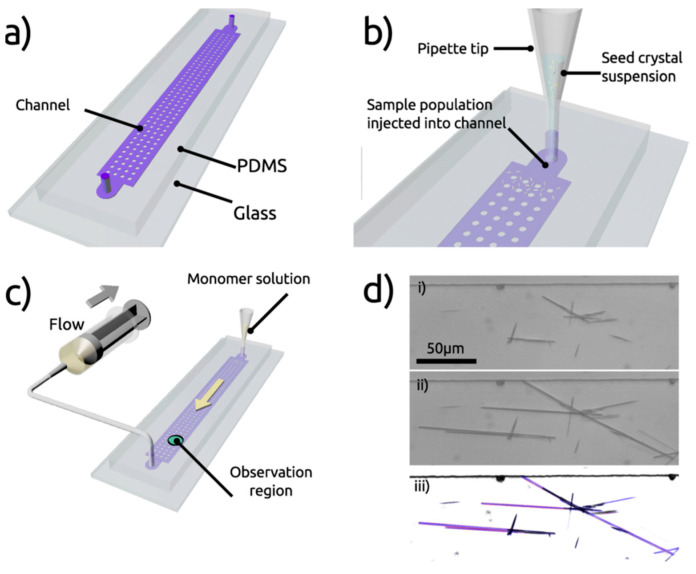
Microfluidics platform for the control of peptide self-assembly. (**a**) presentation of the microre-actor (multiple inlets and central chamber), (**b**) injection of seed crystals, (**c**) injection of a satu-rated solution of FF, (**d**) images via optical microscopy. With permission from Reference [32].

Self-assembly of FF and its kinetics was studied using a microfluidic reactor made by soft lithography, presenting multiple inlets and a large central chamber (Figure 3a). After injection of seed crystals (Figure 3b), a saturated solution of FF was pumped through the reactor (Figure 3c) and a series of images were taken via optical microscopy, in order to measure individual crystalline FF assemblies and to record changes in the dimensions (Figure 3d). The use of microfluidics maintains the system under a non-equilibrium condition, where the crystal dimensions are not driven by the equilibrium solubilities of each face, but by the respective growth rates. This concept would make it possible for peptide self-assembly control through kinetics and not only thermodynamics.

**Figure 4:**
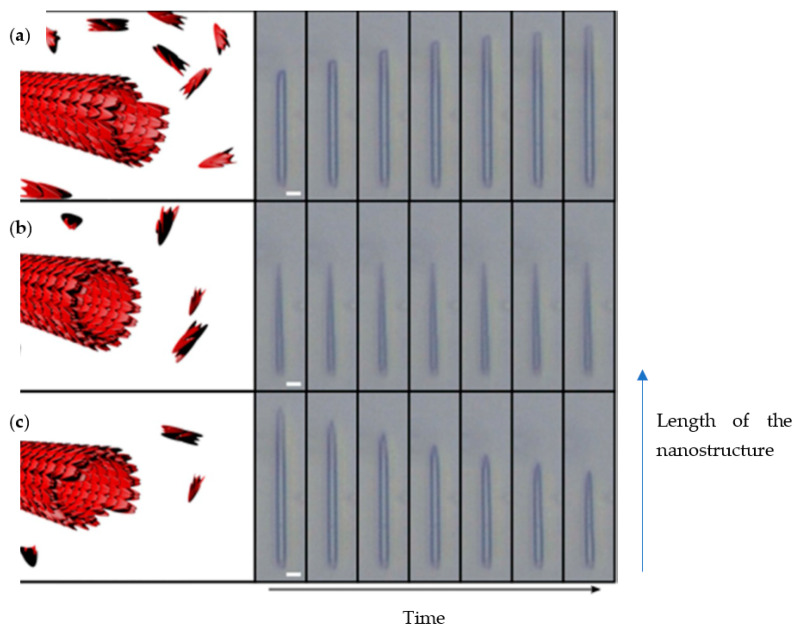
Elongation or shortening of FF nanotubes depending on monomer concentration in microfluidic platform. (**a**) supercritical concentration, (**b**) critical concentration, (**c**) subcritical concentration. Adapted with permission from Reference [33].

The same group further subjected the FF nanotubes to various conditions to demonstrate control of nanoassemblies association and dissociation using a microfluidic device [33]. Firstly, preformed FF nanotubes were pumped into the device so as to be confined by polymethylsiloxane (PDMS) micro pillars. Then FF solutions of subcritical and supercritical building block concentrations were injected into the microfluidic system. The elongation or shortening behaviour of the structures, dependent on the concentration of FF, was examined by light microscopy. The length of the nanotubes was measured at different times to determine the elongation dynamics. At supercritical concentration (3.20 mM) the length of the tubes was increasing over time (Figure 4a), while it remained the same at critical concentration (2.43 mM) (Figure 4b). Using subcritical concentrations (1.60 mM) led to a decrease in the structure length (Figure 4c). As the device was submitted to a constant flow, the concentration of free monomers dissociated from the tubes did not increase, and so therefore the nanotube continued to shorten until complete dissociation.

In conclusion, we have highlighted flow chemistry for linear peptide synthesis, which proved in different aspects (purity, yields, time) to be more efficient than batch classical chemistry. Furthermore, the control of the self-assembly of peptides for the formation of well-designed nanostructures is primordial for future applications. In this context microfluidics appears as a real tool. We can now envisage, thanks to milli- and micro-flow systems, to couple the synthesis and the self-assembly in a dedicated flow device.

## 3. Physico-Chemical Characterization of Short Peptides and Their Assembly

In order to obtain robust nanostructures suitable for biomedical applications, their structure, size, shape, surface chemistry and self-assembly must be optimized. It is therefore essential to control the order in which the amino acids are introduced during the short peptide synthesis and to evaluate their assembly process and physico-chemical properties before testing them for biomedical applications. First, the forces that govern the self-assembly of peptides will be described, followed by a presentation of the nanostructuration in the case of some short peptide families (peptide amphiphiles, aromatic peptides such as diphenylalanine peptide and cyclic peptides). A second part is dedicated to dipeptide and PA characterization by combined classical physico-chemical methods that allow a very deep understanding of the self-assembling process. We will also highlight the interest of complementary methods, based on separation, poorly employed so far for self-assembled peptide nanostructure characterization.

### 3.1. Driving Forces for Self-Assembly

The forces governing the self-assembly of peptides are dictated mainly by two facts, the physical driving forces, and the environmental factors. Here we will describe their implications and effects on peptide self-assemblies.

In equilibrium in an aqueous solution, a peptide molecule and its assemblies adopt the conformation that minimizes their total free energy. The forces involved in the global stabilization of the assemblies through intra- and inter-molecular interactions include mainly hydrogen-bonding, π-π stacking, electrostatic and hydrophobic interactions. These non-covalent interactions are relatively insignificant for isolated peptides, but when they act cooperatively, they can have energies equivalent to a weak covalent bond. Thus, they control the conformation of peptides and dictate the thermodynamically stable supramolecular structures. The type of interaction governing the assembly will depend on the nature of the amino acid sequence or peptide family. Indeed, hydrophobic amino acids that are subdivided in aliphatic (A, V, L, I and M) and aromatic residues (W, Y, and F), provide a general hydrophobic environment and can get involved in π-π stacking, which is of great importance in peptide folding, respectively. Polar amino acids can be involved in hydrogen bounding interactions, either via OH or CONH_2_ groups for uncharged residues (S, T and N, Q, respectively), but also generate specific charge-charge interactions either to stabilize (using opposite charges) or collapse (using equal charges) self-assemblies in the case of charged residues (negatively charged: D and E, or positively charged: K, R and H), respectively. Remaining amino acids that do not belong to the four main aforementioned classes according to the properties of their side chain may offer structural modifications or sites for chemical modifications: G reduces steric hindrance and increases flexibility due to the presence of two hydrogen atoms, P introduces structural rigidity due to the side chain being covalently linked to the amino terminus and C enables disulfide bridges that are of interest for further chemical modifications and inter-peptide crosslinking [34]. It should be mentioned that a process similar to spontaneous protein folding into secondary structures such as α-helix, β-sheet or coiled-coils can be used to drive the self-assembly undergone by peptides in solution [35,36].

The self-assembly process not only depends on the intermolecular interaction between peptide chains, but it is also strongly impacted by the environmental factors. Here we can mention the pH, the ionic strength, the temperature, the concentration of the peptide and the solvent polarity and external stimuli, among others [37]. These environmental factors can affect the equilibria between different structures, or even inside the same structure, the final morphology (e.g., compactness) by altering the interaction forces between peptides and thus triggering a change in the structural conformation of those peptides. In general, changes in the pH and ionic strength of the solubilization medium will alter the electrostatic interactions between side chains and/or between peptides and solvent, whereas modifications in the peptide concentration or solvent nature will predominantly affect Van der Waals and hydrophobic interactions established between self-assembled structures. Several authors have observed the structural changes induced by the pH and played with it to favor the desired structure or to render certain properties to the supramolecular structure with the aim to use it as drug delivery systems. For example, Versluis and coworkers [38] reported the reversible sphere-to-fiber transition of vesicles composed of amphiphilic β-cyclodextrin (β-CD) decorated with adamantane modified octapeptides (LELELELE). In this two-component system three orthogonal interactions are combined: (i) hydrophobic interactions in the cyclodextrin vesicle bilayers; (ii) inclusion complex formation of β-CD and adamantane; (iii) hydrogen bonding in β-sheet domains composed of octapeptide. Using the reversible secondary structure transitions of the octapeptide from random coil to β-sheet domains as a function of pH, a reversible morphological change of the supramolecular complex from vesicles to fibers occurred, thus allowing for a pH-triggered release of the encapsulated contents.

Another important parameter that governs the assembly process is the amino acid sequence and the peptide concentration [39]. Thus, Bowerman [40] and coworkers attempted to elucidate the influence of aromatic amino acids on peptide self-assembly processes. The Ac-(XKXK)_2_-NH_2_ peptide was used to elucidate the relative contributions of π-π versus general hydrophobic interactions to peptide self-assembly in aqueous solutions. Position X was standing for either V, I, F, pentafluoro-F or cyclohexyl-A. At low pH, these peptides remained monomeric because of repulsive forces between protonated K residues. Increasing the solution ionic strength to shield repulsive charge-charge interactions facilitated cross-β fibril formation. As peptide hydrophobicity increased, the required ionic strength to induce self-assembly decreased. Thus, the V sequence failed to assemble in NaCl, whatever the ionic strength was in the range 0 to 1000 mM, whereas β-sheet formation for pentafluoro-F and cyclohexyl-A sequences was observed at only 20 and 60 mM NaCl, respectively. While self-assembly propensity was correlated to peptide hydrophobicity, the presence of aromatic amino acids impacted on the properties of self-assembled structures. Nonaromatic peptides formed fibrils of 3–15 nm in diameter, whereas aromatic peptides formed nanotape or nanoribbon architectures of 3–7 nm widths. In addition, all peptides formed fibrillar hydrogels at sufficient peptide concentrations (8 mM in NaCl solutions of adequate ionic strength to promote prior self-assembly), but nonaromatic peptides formed weak gels, whereas aromatic peptides formed rigid gels.

Eventually, the time left for the peptide to self-assemble is crucial in view of the resulting nanostructure. In this context, Bourbo et al. [41] evidenced that PAs presenting a β-sheet secondary structure self-assembled as spherical micelle-like aggregates while sonicated for 30 min, whereas they rearranged into fibril helical structures when this sonication time increased up to 2 h.

**Figure 5:**
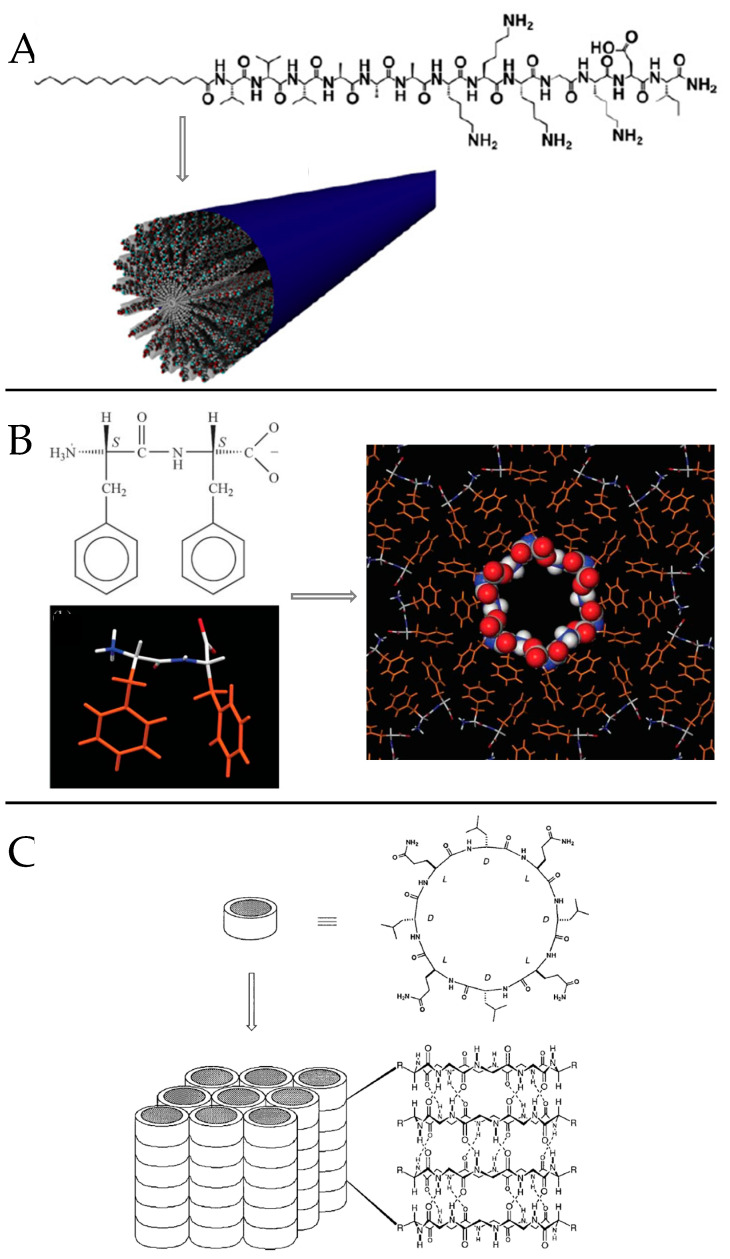
(**A**) Molecular structure of PAs engineered with alkyl tail and scheme of its-self-assembling into stiff cylindrical nanofibers. Adapted with permission from reference [59] (**B**) Molecular structure of FF and model for the construction of hollow FF fibers. Adapted with permission from reference [74]. (**C**) Molecular structure of a cyclic octapeptide with alternat-ing d- and l-amino acids sequence and scheme of its-self-assembling into ordered parallel ar-rays of solid-state nanotubes figuring antiparallel ring stacking and the presence of intermo-lecular hydrogen-bonding interactions. Reproduced with permission from Reference [111].

As mentioned above, peptides self-assemble under mild conditions, into a diverse range of nanostructures that are the result of an intricate and cooperative balance between different intermolecular interactions and environmental factors. Thus, linear peptides and their derivatives often self-assemble into nanostructures, such as nanofibers, nanoribbons, nanotubes, or vesicles; cyclic peptides normally stack into nanotubes and branched peptides often form micelles or vesicle [42,43]. The final sizes of these self-assemblies vary greatly, from a few nanometers to hundreds of microns and the latter nanostructures often template a hierarchy at larger length scales. Among self-assembling peptide systems (Figure 5), we will focus in the following section on short synthetic peptide precursors to give a deeper insight into their engineering strategy, as well as derived nanostructures till the hydrogel state for their interest in nanomedicine.

### 3.2. Short Peptide Families (Self-Assembling Peptides)—Self Assembling Structures

#### 3.2.1. Peptide Amphiphiles (PAs)

PAs are among the most studied self-assembling entities of peptidic nature. These self-assembling peptides have lipid- or surfactant-like characteristics. Their design (Figure 5A) consists in (i) a hydrophobic tail that is composed of nonpolar amino acid residues [44] or aliphatic alkyl C_12_-C_16_ chains [45] or a combination of both, (ii) a linking region that is a short peptide sequence capable of forming intermolecular hydrogen bonding, (iii) a hydrophilic head that contains charged amino acids [44]. for enhanced solubility in water but also for the design of pH and salt-responsive nanostructures and networks, and eventually (iv) a bioactive peptide epitope that can interact with cells [46]. In aqueous medium, hydrophobic interaction is the main driving force governing the self-assembly of nanoarchitectures thus resulting in the sequestration of non-polar groups to form hydrophobic cavities/cores. Hydrophilic end groups are assembled through hydrogen bonding. The balance between hydrophobic interaction and hydrogen bonding drives the self-assembly of peptide amphiphiles. Eventually, electrostatic interactions can enhance the stability of the overall structure when combining PAs of opposite charges [47]. Thus, by rationally controlling the amino acid sequence length and composition, specific nanostructures can be obtained on going from nanofibers and nanoribbons to micelles and vesicles [48,49].

Considering PAs with only amino acids, the tails are normally composed of non-polar amino acid residues. Peptide containing A and V tails were reported to form more homogeneous and stable structures than those made of G, L and I. Such lipid-like peptides can readily self-assemble into dynamic tubular and vesicle structures [50]. The effect of molecular structures was evidenced more deeply on the A_m_K (m = 3, 6 and 9) serie, reporting [51] a decreasing critical aggregation concentration (CAC, down to 15 µM for A_9_K) when increasing the length of the hydrophobic region. The size and shape of the self-assembled nanostructures also changed from unstable plate-like structures (A_3_K) to long nanofibers with uniform diameter of 8 nm (A_6_K) and short nanorods with diameter of 3 nm and length of 100 nm (A_9_K). Increased nanostructure polydispersity was also reported when increasing the tail length of the Glycine rich amphiphilic peptide G_n_D_2_ (*n* = 4 to 10) [52].

Considering PAs engineered with alkyl tail, a minimum of a ten-carbon long aliphatic residue is necessary to induce the hydrophobic effect to cause self-assembly [53]. A dialkyl tail was also reported with two palmitic (C_16_) chains conjugated to a peptide promoting the self-assembly into cylindrical micelles 8.0 ± 2.3 nm in diameter at 2.2 μM CAC [54].

The composition of the peptide segment directly following the hydrophobic region of PA molecules can control the propensity to intermolecular β-sheet hydrogen bonding and thus impact peptide self-assembly and final nanostructuration. Paramonov et al. found that disruption of these hydrogen bonds eliminated the ability of a PA to form elongated cylindrical nanostructures and resulted in spherical micelles [55]. When distressing the hydrogen bonding capacity by alternating hydrophobic and hydrophilic amino acids, an original nanobelt flat architecture was also evidenced [56]. Moreover, the propensity to form β-sheet secondary structures influence the stiffness of nanofiber networks. Stupp and coworkers showed that (i) both the number and fraction of valine residues are especially effective at raising mechanical stiffness whereas alanine residues tend to reduce it [57] and that (ii) the extent of internal order depends on the molecular architecture and peptide sequence of PAs, with branched PAs yielding nanofibers with the lowest degree of internal order [58]. Thus, they further designed stiff nanofibers (Figure 5A) resulting from the ordered arrangement of PA molecules incorporating V and A residues, while the combination of A and G residues resulted in “weaker β-sheet region” promoting soft nanofibers in which PA molecules were more disordered [59].

Amino acids further away from the hydrophobic region play a less important role in stabilizing the supramolecular nanostructure. Thus, functional groups or peptide sequences with specific biological activity in view of specific applications may be introduced with minimal risk of modifying the self-assembly properties of PAs. An extensive variety of peptide epitopes were conjugated to the self-assembling molecules forming bioactive nanostructures. Among them HAV motif in *n*-Cadherin mimetic peptide [60], tenascin-C derived peptide epitope (VFDNFVLK) [61] or laminin- [62,63] and BDNF-derived [64] peptides (containing IKVAV and RKKADP sequences, respectively) were successfully developed, but also systems displaying RGDS [65], FGF-2 [66] and VEGF [67]. Such epitopes are presented on the outer surface of the supramolecular nanostructures. A length tuned G linker (*n* = 1 to 5) is systematically used to space it from the nanocarrier, the longest linker leading to the strongest bioactivity [48]. Most of the time, mimetic peptides must be combined with a well-known self-assembling PA, such as Lauryl-VVAGE(E-Am), to enable the supramolecular nanoorganization an create bioactive architectures [60].

Eventually, the morphology of PAs can also affect their self-assembly. It is to be mentioned the case of cone-shaped peptides, such as Ac-GAVILRR-NH_2_, that bear a large cationic head and self-assemble into spherical micelles (CAC values: 0.82 mM in water and 0.45 mM in phosphate buffer solution, PBS) to further give rise to nanodonut-shaped structures when the peptide concentration increases, due to the geometry restrictions of the cone-shaped peptide [68].

Most of the above mentioned fibrillar structures are reported to form hydrogels at sufficient peptide concentrations. This sol-to-gel transition is triggered by a change in the solution conditions, among which pH modification and addition of salt (divalent ion, such as Ca^2+^), due to the importance of charge-charge interactions in the self-assembling process. Thus, self-assembly could be induced reversibly by changing the pH of the PA solution [53]. An alternate method to control the self-assembly without disturbing the solution is the use of an external stimulus such as light. PA molecule containing a 2-nitrobenzyl group positioned on the β-sheet forming sequence is reported to undergo a sol-to-gel transition in response photocleavage at 350 nm [69]. Light-responsive bioactive materials can also be designed when integrating the photolabile group on the peptide backbone at the epitope linker site. As proof of concept, a photocleavable nitrobenzyl ester group was used to enable the light-triggered removal of the RGDS cell adhesion epitope from PA molecules without affecting self-assembling peptide scaffolds [70].

#### 3.2.2. Aromatic Peptides and the Particular Case of the Diphenylalanine Peptide (l-Phe-l-Phe, FF)

FF is the simplest aromatic peptide capable of forming π-π stacking interactions between aromatic rings to spontaneously form thermodynamically stable nanostructures. By extension, the so-called aromatic peptides designate a family of peptides consisting in a hydrophobic aromatic end group and a relatively short hydrophilic peptide segment [71].

FF is the core recognition element of the Alzheimer’s β-amyloid polypeptide. The pioneering work of Gazit and coworkers [72] highlighted its self-assembling properties to form discrete, stiff, and well-ordered water-soluble peptide nanotubes with about hundred nanometers diameter, several microns length and of remarkable physical and chemical stability [73]. Supramolecular interactions between COO^−^ and NH_3_^+^ groups are at the origin of head-to-tail-interactions between dipeptides. Resulting cylinders of peptide main chains interact with one another by π-π stacking of phenyl rings present in hydrophobic side chains, thus promoting the formation of pH-responsive fiber (Figure 5B) [74]. Such orientationally aligned nanotubes may further assemble into microtubes increasing the FF concentration from 1 to 2 mg·mL^−1^ [75]. Control of the assembly dimensions and morphology can also be induced by varying the solvent nature [76,77]. The same group evidenced that GFF, a highly similar analogue to FF peptide, forms stable spherical nanometric assemblies. The introduction of a thiol group by incorporation of a C residue into FF peptide modified its self-assembly properties resulting as well in spherical vesicles instead of nanotubes [78]. Eventually, the IF dipeptide was evidenced to self-assemble in aqueous solution, leading to a thermoreversible and transparent gel consisting in nanostructured fibrillar networks. IF exhibits a solid state at 293 K while it is fluid above 313 K, its transition temperature depending on the peptide concentration (304 K and 299 K for a 2 and 1.5 %, *w*/*v*, respectively) [79].

To a larger extent the coupling of aromatic groups to short peptides may induce self-assembly by promoting π-π stacking. Among them naphthyl (Nap) [80,81,82] and pyridyl (Pyr) [83,84] groups, phenylboronic acid [85] or fluorenylmethoxycarboenyl (Fmoc) when introduced to the N-terminus of the peptide segment act as gelators. Among them, Fmoc group has been widely studied. Fmoc-LD peptide molecule and other related dipeptides were the first studied [86]. Later Fmoc-FF was described to self-assemble in an anti-parallel β-sheet pattern with backbone hydrogen bonding between the peptides, while the aromatic Fmoc units form π-stacked aggregates creating a cylindrical structure [87]. More systematic studies were reported by Xu’s [88] and Ulijn’s [87]. groups on the self-assembling properties of Fmoc-bonded dipeptides, highlighting that all dipeptides, except GT and GF, can auto-organize into pH-responsive nanofibers in aqueous solution. A similar architecture was observed for Fmoc-LLL, but with a larger diameter [89]. More designs of Fmoc-bonded tripeptides (Fmoc-FWX and Fmoc-FFX, X = H, R, S, E or D) were proposed by He’s [90,91] group to form handedness-tunable nanohelices. The Fmoc-FW was reported to self-assemble into right-handed nanohelices, while the Fmoc-FF derivatives tended to form left-handed nanohelices. Dimension and morphology of the nanostructures of the self-assembled nanohelices were thus dependent on the nature of the terminal residues X and the aromatic side chains at the second residues (W or F) with regards to the diameter and handedness, respectively.

Co-assembly of FF with FF derivatives or other functional materials was extensively explored to design unique multicomponent supramolecular materials [92,93]. For instance, the co-assembly of FF and Boc-FF (Boc = tert-butoxycarbonyle) in various ratios provided a variety of “biomolecular necklaces” consisting of spherical assemblies connected by elongated elements [94]. The co-assembly of FF with Fmoc-l-DOPA(acetonated)-d-Phe-OMe (DOPA = 3,4-dihydroxyphenylalanine) yielded different nanostructures [95] depending on the concentration of the two peptides: oval biconcave disks, similar to red blood cells, were observed at 1 mg·mL^−1^, while spherical structures with bulges on their outer surface, similar to white blood cells, where obtained at higher concentrations up to 2 mg·mL^−1^. Thus, morphology and physical properties of co-assembled supramolecular structures showed a strong dependence on the ratio of precursor peptides.

Supramolecular hydrogels could also be architectured from aromatic peptides with tunable stiffness by varying the co-assembly concentrations [96] and nature. For example, while co-assembling peptide-based gelators Fmoc-YL or Pyr-YL with surfactant-like molecules Fmoc-S or Pyr-S, the S residue presented carboxylate functionality to the surface of the fibers [97], enabling subsequent cross-linking upon exposure to divalent cations such as Ca^2+^. Such supramolecular hydrogel formation can be regulated by different external stimuli. Reversal of hydrophobicity by means of Y phosphorylation/dephosphorylation was reported by Xu’s and coworkers [98,99,100,101] that designed and synthesized a new hydrogelator Nap−FFGEY. A kinase/phosphatase switch was used to control the phosphorylation (Nap-FFGEY-P(O)(OH)_2_) and dephosphorylation (Nap−FFGEY) of the hydrogelator, thus inducing the collapse or restauration of the nanofiber assembly, respectively. Another strategy to trigger self-assembly of peptide hydrogels, besides solvent composition and pH variation [102,103,104], is enzymolysis [105,106,107,108]. Ulijn’s laboratory [109] condensed Fmoc-F and FF segments enzymatically using thermolysin to form a hydrogel of Fmoc-FFF by performing reverse hydrolysis.

#### 3.2.3. Cyclic Peptides

Cyclic peptides are rationally designed heterochiral ring-shaped peptides subunits forming β-sheet-like structures. They self-assemble by stacking to mainly form open nanotubes and intermolecular hydrogen bounding participate in stabilizing the nanostructure The cyclic heterochiral octapeptide cyclo [D-AL-ED-AL-Q)_2_] was the first sequence described by Ghadiri and coworkers [110]. By alternating D and L-amino acids the investigators generated a planar ring with a specific diameter (7–8 Å), the later internal diameter being controlled by changing the number of amino acids in the peptide sequence (Figure 5C) [111]. The authors hypothesized that cyclic peptide having an even number of alternating D-and L-amino acids could adopt a low-energy ring shaped flat conformation, all backbone peptide amide groups being orientated perpendicular to the plane of the structure, and participate in backbone-backbone intermolecular hydrogen bonding to generate an adjacent antiparallel β-sheet structure [110]. From molecular modeling and experimental results cyclic octapeptides were found to present the optimum balance between a low ring strain structure and a flat ring-shaped conformational state. Four new peptide nanotube solid-state ensembles where further described and characterized: cyclo [(L-QD-A)_4_-], cyclo [(L-QD-L)_4_-], and cyclo [(L-QD-F)_4_-] subunits giving rise to highly ordered nanotube crystals, while cyclo [(L-QD-V)_4_-] only formed semicrystalline needles [111].

Ring stacking is favored at acidic pH, due to weakened intermolecular repulsive electrostatic interactions and strengthened attractive side chain/side chain hydrogen bonding. Ghadiri and coworkers exploited the ionization state of the carboxylic acid moiety in E residue side chain to trigger the phase transition toward self-assembled nanotube particles that tightly pack into rod-shaped crystals [111]. By modifying the side chain of K residues with cationic 1,4,5,8-naphthalenetetracarboxylic diimide the same group also engineered a cyclic heterochiral octapeptide that undergoes redoxtriggered self-assembly in aqueous solution into peptide nanotubes [112].

Another cyclic peptide that was reported in the literature was the Lanreotide cyclic octapeptide (d-nalphtyl-ACYD-WKVCT-CONH_2_), which was synthesized as a growth hormone inhibitor [113]. Lanreotide was shown to self-assemble into nanotubes of viral capsid-like dimension, that are of remarkably monodisperse diameter (24 nm). Peptide dimer building blocks self-assembled into antiparallel β-sheets through an alternating pattern of the aliphatic and aromatic amino acid residues to form helicoidal filaments that build the nanotube walls [114].

### 3.3. Characterization Methodologies

Characterizations of self-assembled peptide nanostructures allow to determine their global structure (peptidic sequence, size, diameter, charge density, shape, CAC), their architecture (secondary, tertiary and quaternary structure) and help for understanding the mechanism of self-assembly and the influence of some external stimuli (pH, salts, temperature...). These characterizations are of importance for biomedical applications, so as to design the most biocompatible and biodistributable nanostructures with expected properties in biological conditions. Due to their dynamic self-assembly processes, multiple characterization methods have to be implemented jointly, allowing for large time and length scales. The classical methods employed so far are either spectroscopic (nuclear magnetic resonance (NMR), infra-red (IR), Raman, fluorescence, circular dichroism (CD)), scattering (X-ray diffraction, small-angle X-ray scattering (SAXS), dynamic light scattering (DLS)) or microscopic (transmission electron microscopy (TEM), scanning electro microscopy (SEM), atomic force microscopy (AFM), fluorescence microscopy) analysis. In some cases, computational and theoretical approaches are complemented to help for the rationalization of the experimental results and help for self-assembling mechanism elucidation [115].

Some recent reviews describe deeply these classical methods for characterization of self-assembled peptide nanomaterials, among which in the context of functional materials in information technologies and environmental applications [37], or biomedicine and biotechnology [35]. They highlight their performances as well as the need for advanced sample preparation and sophisticated analysis tools.

Here, we will provide a non-exhaustive review of the literature, presenting the interest of some combined classical characterization methods of small peptides (dipeptides, short peptides, amphiphilic peptides) of varying composition, in order to elucidate their structure, their interaction with their environment or the effect of stimuli due to experimental conditions. We will also highlight the potential of complementary methods, based on separation, poorly employed so far for self-assembled peptide nanostructure characterization.

#### 3.3.1. Dipeptide and PAs Characterization by Combined Spectroscopic, Microscopic and Scattering Methods

Self-assembly morphology and geometric parameters (size, diameter) can be determined by imaging methods (SEM, TEM, AFM, optical microscopy) [35] focusing also on physical properties, such as thermal, chemical, and conformational stability [116], rather than structural organization. The secondary structure (α-helix, β-sheet, random-coiled conformation, in a 5 nm range) can be obtained by spectroscopic methods (CD, NMR, Fourier-Transform IR (FTIR)) thanks to the understanding of the bond properties, vibrational modes and covalent and noncovalent interactions of the peptide structures. Although widely used, scattering methods need complex interpretation. The analysis can be performed in solution or in the solid-state, which could in this case modify their architecture. Furthermore, some limitations appear when applied to peptide nanotubes, as evidenced for X-ray crystallography and solution NMR, because of their large molecular weight, non-crystalline and sometimes insoluble nature and in the context of stimuli-responsive dynamic architectures [117].

Some literature presents the characterization of dipeptides for the elucidation of their self-assembly architecture or co-assembled nanostructures between two different dipeptides, as well as the influence of solubilization medium, or the evidence of complex structures with other compounds (PNA or NPs).

Di-D-diphenylalanine (di-D-FF) nanotubes were explored by polarized vibrational spectroscopy, which can elucidate the structure of nanoobjects through the information about the orientation of chemical groups in an anisotropic sample. They evidenced that the nanotube had cylindrical symmetry with different oriented functional groups to the nanotube axis: a parallel orientation of the C–N bond of CNH_3_^+^ groups, a perpendicular one for COO^-^ groups, and a 54° angle orientation for the peptides’ carbonyl groups. This data allowed to confirm the unique orientation of the di-D-FF molecules with respect to the nanotube main axis [117].

**Figure 6:**
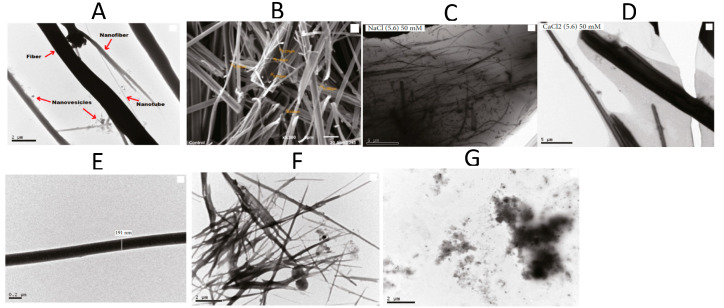
Microscopy images of FF nanostructures. TEM (**A**) and SEM (**B**) in water (control sample). TEM in 50 mM NaCl (**C**) and 50 mM CaCl_2_ (**D**) at pH 5.6. TEM at pH = 3 (**E**), pH = 5 (**F**), and pH = 10 (**G**). Adapted with permission from Reference [26].

TEM, SEM, and FTIR spectroscopies were used to characterize the nanotubes formed by FF in different salt solutions (NaCl, CaCl_2_, and AlCl_3_), concentrations (50, 100 and 200 mM), and pH (3 to 10) (Figure 6). FF nanotube formation through self-assembly was identified as a delicate balance between electrostatic, hydrogen bonding and hydrophobic interactions, whose modifications can impede nanotube formation. The presence of NaCl and CaCl_2_ salts contributes to the self-assembly of very long FF nanotubes agglomerates in water, due to a combined screening effect and the fact that cations are structure-forming and promote hydrophobic interactions. Salt bridges between either C-termini and/or N-termini appear as alternatives to peptide bonds, and can lead to radial and longitudinal nanotube growth. In the presence of AlCl_3_, the authors evidenced an imbalance due to the excess of Cl^−^, impeding the nanostructure formation [26].

Another dipeptide (LF) self-assembly was explored by ^13^C and ^15^N solid-state NMR spectra [118]. Solid-state NMR helps for understanding structural conformations and dynamics of a variety of solid samples. Whereas ^13^C NMR chemical shift values provide information on secondary structures, ^15^N NMR signals can determine hydrogen bond strengths, which can help for understanding the mechanisms of peptide self-assembly. In this context, the morphology of self-assembled LF peptides in an ethyl acetate-hexane solution was first determined by SEM, highlighting a straight nanofiber (80 nm width). Then, well resolved ^13^C and ^15^N solid-state NMR signals of LF peptide in the nanofiber were shown in good correlation with the calculated ones for the crystal structure via the density functional theory (DFT). Therefore solid-state NMR structural analysis combined with DFT calculations of self-assembled dipeptides is an effective approach.

Ordered structures in the self-assembly of homoaromatic peptides were demonstrated in a mixture of two homoaromatic peptides [119]: the FF peptide which self-assembles to form tubular assemblies and its Boc-protected analogue (Boc-FF) forming either spheres or fibers depending on the solvent. Their co-assembly was studied by SEM, AFM and FTIR. The formation of peptide-based spherical assemblies (1–4 μm diameter) connected by elongated structures (~300–800 nm) in a three-dimensional arrangement were evidenced by SEM and AFM. FTIR spectra of the co-assembled structure differed from the one of each self-assembled structure, highlighting an α-helix structure (1653 cm^−1^) along with a β-turn conformation (1684 cm^−1^), in a unique structure, that was designated by the authors as «biomolecular necklaces».

A more complex nanostructure made of FF hybrids with peptide nucleic acids (PNA) was studied by FTIR and fluorescence [120]. Critical agregation concentration determination via fluorescence measurements showed that the conjugation of one or two PNA monomers to the well-known aggregating motif FF improves their tendency to self-assemble. FF was shown to drive the aggregation process, through π-π stacking between the phenyl rings, but hydrogen bonds between PNA were also visualized, depending on the nature and number of bases.

Finally, the interest of FF nanostructures as potential surface-enhanced Raman spectroscopy (SERS) substrates for biosensing and biomedical applications was demonstrated via TEM, Raman, luminescence and DFT calculations [121]. At 150 °C, L,L–FF micro-nanostructures (FF-MNSs) are subjected to an irreversible phase transition from hexagonally packed (hex) micro-nanotubes to an orthorhombic (ort) structure. In these two phases, FF-MNSs organize Ag and Au nanoparticles (NPs): the metal NPs form chains on hex FF-MNSs as inferred from TEM images and a uniform non-aggregated distribution in the ort phase. These structures were therefore potential substrates for the SERS, with an activity of the ort phase twice higher, in the case of Au NP.

PAs are of high interest for various applications, and were characterized for the understanding of the impact of sequence nature or end-capping on the structures, as well as the influence of the experimental conditions.

MK Bauman et al. demonstrated the influence of primary and secondary structure designs on supramolecular assembly of PAs: L6K2, I6K2 and V6K2 [39]. While varying the apolar tail amino acids, CD spectroscopy allowed to determine the secondary structures, with a β-sheet structure for short amphiphilic diblock peptides coding the hydrophobic tail (I6K2), while the absence of β-sheet hydrogen bonding for L6K2 and V6K2 yielded micellar rods. Combined TEM and AFM analysis demonstrated flexibility of the structure: different sizes were obtained, in the 100–600 nm and 80–360 nm ranges, at 0.01 mM and 0.1 mM peptide concentrations, respectively, with sheet height in the 2 to 4 nm range, agreeing with the length of one to two monomers. Therefore, the nature of the amino acids in the sequence and the peptide concentration have an impact on the agregate shape and width and can tune average rod length and ribbon/sheet area over a large range. Thus, the main differences in macromolecular structures seem to arise from the ability to form β-sheet interactions between the monomers.

The formation of β-sheet secondary structures for two amphiphilic peptides (Ar-EFEFACEFEFEP and Ar-EFEFEFEFEFEP, Ar = 4-acetamidobenzoate) was characterized by V. Bourno et al. [41] They are composed of repetitive dyads of hydrophilic and hydrophobic amino acid residues, and differing in the absence of the AC dyad for the latter peptide. P residues were incorporated to enhance the formation of ordered β-sheet assemblies. By measuring the molar ellipticity at 216 nm, CD evidenced first a clear signature of β-sheet formation, that after 20 min led to a transition between different forms of β-sheet containing structures. The self-assembly process was further studied with the fluorescence-based assay with Thioflavin T (ThT): the ThT interactions with β-sheets was observed at 480 nm, with a non linear increase with time, reflecting expansion of the sheets together with changes in their morphology. Finally AFM showed the formation of spherical micelle-type aggregates after a relatively short time of assembly, followed by a rearrangement into fibril helical structures, of several micro-meters in length, 20–30 nm in width, and 14.5 ± 2.5 nm in helix pitch.

The synthesis and characterization of a novel amphiphilic peptide KA6 was performed by CAC determination (absorbance at 470 nm), zeta-potential measurement, AFM images and CD for secondary structure [22]. This peptide formed of a hydrophobic chain of six A residues contains a carboxylic group on the C-terminus (pKa 3.2) and two amino groups on the N-terminus and the LK side chain (pKa 7.9 and 10.5, respectively). It was confirmed that electrostatic interaction mainly governs its self-assembly, as a pH shift leads to a change in both the CAC value and the micellar structure. At pH 2, positively charged micelles (+29 mV zeta potential) were evidenced above a CAC of around 0.5 mM, in tubular and tubular balloon-like micellar structures. Some micelle fusion could be also evidenced from AFM images. The structures were of 10–15 nm height (multilayer assembly), 30 nm or 50 nm width, for tubular and herring bone types, respectively. At pH 7, positive micelles (+24 mV zeta potential, CAC of around 1 mM) form similar tubular type micelles (8–10 nm height, 15–20 nm width) and thin needle-like structures (1.5–2 nm height), as evidenced by AFM and CD. At this pH the self-assembly is governed by the positive amino groups. At a pH above their pKa, the micellar structure inverts exposing the opposite end of the peptide chain to the solution, leading to negatively charged micelles (−41 mV zeta potential) formed above 2 mM, with a β-sheet like CD spectrum. The clear dipolar behaviour of the KA6 peptide leads to a drastic modification of the supra molecular assembly, solely due to electrostatic interaction tuning, which highlights its potential for pH-sensitive materials.

**Figure 7:**
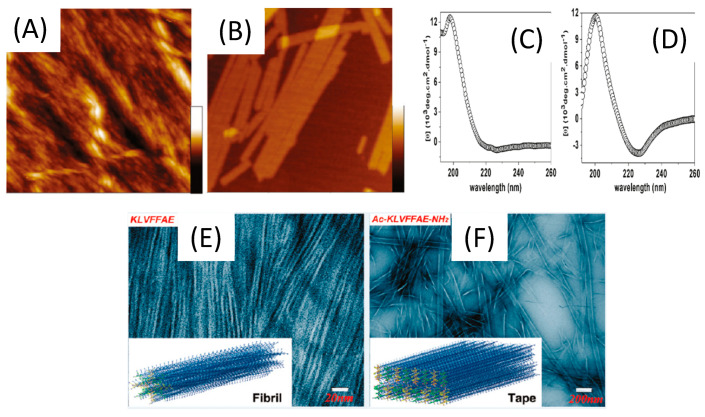
Characterization of short β-amyloid (Aβ) peptides (16–22) at a concentration of 3.0 mM, in acetonitrile/water (2:3, *v*/*v*) at pH 2.0. AFM topographical images of KLVFFAE (**A**) and Ac-KLVFFAE-NH2 (**B**). All images are 1.5 × 1.5 μm in size, and the height scales are 70 and 15 nm for (**A**) and (**B**), respectively. CD spectra of KLVFFAE (**C**) and Ac-KLVFFAE-NH_2_ (**D**). TEM micrographs of aggregated structures of KLVFFAE (**E**) and Ac-KLVFFAE-NH2 (**F**), Inserts: schematic representation of a model nanofibril formed by KLVFFAE and a model nanotape formed by Ac-KLVFFAE-NH2 in these experimental conditions. Adapted with permission from Reference [122].

The characterization and comparison of two short β-amyloid (Aβ) peptides (16–22), KLVFFAE and Ac-KLVFFAE-NH_2_ showed the influence of terminal capping to the molecular structure and electrostatic interactions [122] (Figure 7). At acidic pH, combined TEM, AFM and CD analysis revealed that the uncapped peptide self-assembles in straight nanofibrils of 3.8 ± 1.0 nm, whereas the capped one was structured in nanotapes with a width of 70.0 ± 25.0 nm. While both aggregates form β-sheet structures, CD along with fluorescence and rheology measurements indicated weaker hydrogen bonding and weaker π-π stacking for the uncapped one. This is consistent with the strong electrostatic repulsion due to the two positive charges at the N-terminus of uncapped KLVFFAE. Capping the peptide termini contributes to a strong reduction of this repulsion (one positive charge) and favors stronger lamellar structuring, leading to the formation of rather flat nanotapes. The same study was performed at neutral and basic pH, evidencing the change in morphology of self-assembled Ac-KLVFFAE-NH_2_ to nanofibrils at neutral pH (pH 7.0) to nanotapes at alkali pH values (12.0) where the charge on the capping end was reversed. Therefore, capping the peptide termini results in the tuning of intermolecular electrostatic interactions upon self-assembly, inducing different morphologies according to pH.

#### 3.3.2. Emerging Separation Methods for Peptide Nanostructure Characterization

As indicated previously, few separative methods were described for self-assembled peptide nanostructure characterization, i.e., ion mobility and electrophoresis. Electrokinetic separations are powerful characterization methods of biological compounds, among which peptides, without sophisticated sample preparation. In their different modes (zone electrophoresis, isotachophoresis, gel electrophoresis, isoelectric focusing, affinity electrophoresis, electrokinetic chromatography, frontal and hybrid modes) and formats (classical of microchip), they provide information on purity, physico-chemical and biochemical characterizations of peptides [123]. As the background electrolytes (BGE) can be of a broad variety, from aqueous to non-aqueous media, with a wide range of buffering salts and additives, pHs and ionic strengths, they are sufficiently versatile for allowing the solubilization and separation of various peptidic structures, while requiring very small amount of sample (in both formats). Furthermore, electrokinetic separations can be coupled to different detectors, i.e., UV, fluorescence, chemiluminescence, electrochemical, electrochemiluminescence, MS, NMR and IR spectroscopy. Therefore, electrokinetic methods seem promising for a deep physico-chemical characterization of peptides and their assembly, as they allow determining physicochemical characteristics such as effective charge, pI, Mr, Stokes radii, diffusion coefficients, acidity (ionization) constants (pKa) of ionogenic groups, and binding (association, stability, formation, dissociation). Electrophoretic mobility shifts can evidence a change in structure and assembly of peptidic sequences, in an equilibrium state, while varying the BGE nature. This could help for mapping an equilibrium diagram of the structure of self-assembled peptides in different biological media. We present here some articles highlighting the potential of separations, either with simple UV or MS detection, for purity and sequence determination as well as interaction studies. As some of the peptidic sequences are hydrophobic, separation conditions in hydro-organic or organic media were developed.

Water insoluble cyclic peptide [Gly6]-antamanide and its complexes with sodium and potassium ions were successfully separated in non-aqueous media (methanolic BGE) [124]. The capillary electrophoresis (CE) affinity mode allowed to quantify the apparent binding constant of [Gly^6^]AA [Gly^6^]AA with both Na^+^ and K^+^ (26 ± 1 and 14 ± 1 L/mol, respectively). These experimental data were complemented with density functional theory (DFT) calculations, to calculate the interaction energies of the [Gly^6^]AA-Na^+^ and [Gly^6^]AA-K^+^ complexes (−466.3 and −345.2 kJ/mol, respectively), and evidence the position of both cations in the cavity of the peptide along with the interatomic distances within the complexes.

**Figure 8:**
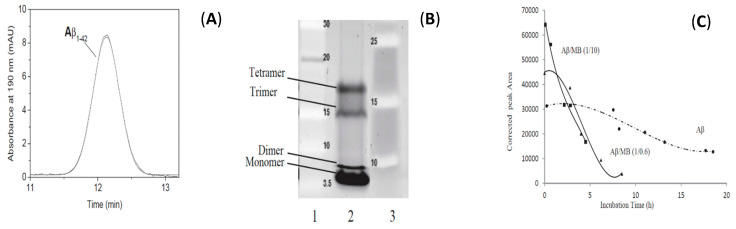
Electrophoretic study of Aβ_1–42_ sample. (**A**) TDA (UV Trace) (**B**) Tris-glycine-17%-SDS-Page analysis. (**C**) Corrected absolute peak areas over time of the electrophoretic profiles of Aβ_1–42_ in presence (two ratios) or absence of Methylene Blue. Adapted with permission from Reference [125].

Another CE separation performed in aqueous BGE allowed to follow the in-vitro oligomerization (or antioligomerization) process of the 42 amino acids amyloid β-peptide (Aβ_1–42_) that can lead to neurotoxic oligomers (less than 50 kDa) [125]. They first developed a simple, fast and reproducible sample preparation of Aβ_1–42_, which allows obtaining this peptide mainly in its monomeric state. They evidenced by CE a mobility shift from the monomer to the formation of four self-assembled structures, and could monitor in real time the oligomerization kinetics. Taylor Dispersion Analysis in its CE format allowed to estimate the size of the very early formed structures (1.8 nm) and gel electrophoresis (SDS-PAGE) showed the predominance of the monomer. A kinetic study of the oligomerization of Aβ_1–42_ with or without the addition in the sample of Methylene Blue (MB), an anti-Alzheimer disease candidate, was then performed by CE. MB, reported to inhibit Aβ_1–42_ oligomerization, was shown to modify the oligomerization by promoting fibrilization (Figure 8).

So as to improve the characterization, few articles coupled CE to, MS which is a classical detector in proteomics for peptide determination. Cortez et al. designed, synthesized and characterized new cyclic d,l-α-alternate amino acid peptides that could further provide PNTs of various properties [7]. In an hydro-organic BGE (H_2_O/EtOH 50:50, *v*/*v*), they coupled CE to electrospray ionization mass spectrometry in positive mode for an efficient characterization. From the eight original CP sequences of 8, 10, and 12 d,l-α-alternate amino acids (with a controlled internal diameter from 7 to 13 Å), the mass spectra of each separated electrophoretic peak evidenced the efficient sequence synthesis and peptide cyclization without residual corresponding linear, protected, or partially deprotected peptides, thereby proving their purity. In most cases, they presented electrophoretic mobilities in accordance with their global charge and mass. However unexpected behaviors appeared, that could evidence specific cyclic configurations of the peptides due to their sequence nature.

A nonaqueous CE-MS (NACE-MS) method was developed to separate and characterize highly hydrophobic temporin peptides (in the 1350 to 1400 Mr range). The mass spectrometer offered a second dimension of separation for peptides having identical migration times but different structures [126].

A comparative study by CE and ion mobility spectroscopy (IMS) was performed for following folding transition of a 13-mer polyproline peptide (from the all-cis polyproline I to the all-trans polyproline II) conformation upon immersion in an aqueous solvent [127]. Synchroneous folding processes were evidenced by both methods. From the eight conformers observed using ion mobility, only five peaks were observed in CE, which can be modeled as sums of the observed IMS conformers, proving the first direct evidence that multiple folding intermediates are present in solution.

IMS coupled to MS was used to reveal the peptide self-assembly mechanism during amyloid fibril formation. The authors studied a series of amyloid-forming peptides clipped from larger peptides or proteins associated with disease. IMS-MS allowed to study the structural evolution of soluble peptide oligomers one monomer at a time [128]. IMS-MS analysis of peptide self-assembly thus opens new avenues for the investigation, detection and eventual treatment of pathogenic processes in amyloid diseases implicated by soluble peptide or protein oligomers.

These few works combining separation methods with UV or MS detection highlight the interest for such methodological developments, that could also be further coupled to NMR. They would provide in one single experiment various information, going from purity, size, charge, sequence determination, interactions and the identification and control of self-assembled nanostructures, in any type of medium mimicking biological conditions, so as to employ them for biomedical applications.

## 4. Biological Evaluation and Applications in the Biomedical Field

The use of materials (biological, inorganic, organic or hybrid) at the nanolength scale has revolutionized the biomedical field by means of the so-called nanomedicine spanning many areas such as drug delivery, diagnosis and imaging tools, therapeutics, regenerative medicine, and tissue engineering, among others [129]. In this scenario peptides with the ability to self-assemble are gaining much attention thanks to already mentioned features of biocompatibility, easy way of design and synthesis, versatility of structures and functions, stability, and capability of being stimuli responsive. In this section recent advances in the application of peptide-based nanostructures for biomedical purposes will be discussed. Two main issues will be afforded: (i) biological tests commonly used for the validation of peptide nanostructures for biomedical applications and (ii) biomedical applications of such structures going from the development of diagnostic tools to in vivo applications for the monitoring, follow-up, and treatment of diseases (e.g., cancer).

### 4.1. Biological Tests for Validation of Self-Assembled Peptide Nanostructures

Before the development of any in vivo application and clinical translation of any new product a series of biological tests should be done to ensure the safety, figure out the mechanism of interaction and uptake by cells, tissues and organs, evaluation of the fate, clearance, or bioaccumulation pathways, etc. [130]. A large panel of in vitro and in vivo evaluations is available, their selection is not trivial and could vary as function of the envisioned application. In this section we will report the main in vitro and in vivo preclinical tests allowing the full characterization of self-assembled peptide nanostructures for their further application in the biomedical field.

#### 4.1.1. In-Vitro Tests

The assessment of hazards and risks of new products by the in-vitro tests is a crucial step towards the in-vivo application and clinical translation. These tests not only ensure the safety of the proposed product but also are the first proof of concept on the feasibility of the envisioned product application. Thus, a wide panel of assays is available, and the selection of appropriate evaluations is not trivial. After a pertinent physicochemical characterization by different analytical methods (microscopic, spectroscopic, scattering, and hyphenated ones, as well as in silico studies) biological tests are mandatory. In contact with a physiological environment, their “synthetic identity” and properties could indeed be affected and modified, leading to a “biological identity” with specific physiological responses. As these innovative nanostructures for nanomedicines or nanotherapies are quite recent, their nanosafety has remained poorly evaluated mainly because the conventional approaches are not well-adapted to the nanosize characterization and the lack of specific standards and protocols [131]. In this view a recent review focuses on proposed guidelines for the evaluation of their biological activity, toxicological effects, nanomaterial-cell interaction and uptake mechanisms, and test endpoints based on the analysis of an extensive literature in the field (more than 200 works) [132]. They have identified that the main processes for nanomaterials to cause cellular damages are via oxidative stress leading to pro-inflammatory effects, fiber paradigm for those nanomaterials presenting nanofiber structures causing granulomas in the peritoneal cavity and lung, genotoxicity and/or release of toxic ions or molecules from their structure. They therefore propose different cellular evaluations: (i) cytotoxicity evaluation via (a) the visualization of cellular morphological alteration, the assessment of plasma membrane integrity, and the cellular metabolism (mitochondrial activity, the well-known MTT test), (b) cell death evaluation (apoptosis or necrosis), (c) cell proliferation, and (d) epithelial cell barrier damage (cell adhesion); (ii) Oxidative stress evaluation (detection of reactive oxygen species, ROS); (iii) Pro-inflammatory reactions evaluation via the analysis of related proteins (cytokines, chemokines, and growth factors) by enzyme-linked immunosorbent assay (ELISA) tests; (iv) genotoxicity evaluation, including DNA lesions, chromosome aberrations and mutations determined via Comet and micronucleus assays in mammalian cells. Finally, in the report of Fabbrizi et al. [133] the importance of the cell line (primary vs. immortalized cells), type of system (2D vs. 3D), model of culture (co-culture, perfusion, single cell) selection is discussed. Another important aspect is the tendency to go towards the development of methodologies for the assessment of risks and hazards based in the 3Rs (replacement, reduction, and refinement) limiting the in vivo tests and animal experimentations and searching for alternatives such as 3D cultures, human primary cells and organoids approaches [131,132,133].

FF, one of the most studied dipeptides forming nanostructures for a wide range of biomedical applications, has been tested with several in vitro tests. In the work of Silva et al. [134] different in vitro tests were performed for the evaluation of the microtubes formed by the self-assembly of the FF in view of their use as nanocarriers for drug delivery. The cytotoxicity test showed a good cell viability (>60%) up to 5 mg·mL^−1^ peptide concentration. More interestingly, the authors performed a hemolytic assay to figure out the toxicity of FF microtubes, via membrane interactions studies. The results showed that the hemolytic behavior of FF microtubes were similar to that of well-described and low-toxic nanocarriers, such as cyclodextrins and polymeric nanocapsules, inducing the hemolysis at concentrations higher than 2.8 mg·mL^−1^. They further developed different formulations of FF with phthalocyanines as photosensitizers in the treatment of cancer by phototherapy [135]. The in vitro tests showed that the presence of the FF nanotubes enhances the antitumor activity of the phthalocyanines photosensitizer because it facilitates and improves around three times the uptake of the phthalocyanine by cells. They also evidenced that the FF nanostructure alone does not induce any apoptosis (<15%) having a good cell viability (by the neutral red uptake assay and an annexin V-fluorescein isothiocyanate (FITC)/iodide propidium double staining flow cytometric analysis) at 0.2 mg·mL^−1^. On the contrary when they were associated to the phthalocyanine more than 80% of cell death was observed.

The hydrogel state of peptide self-assemblies presents a high biocompatibility assessed by seeding and viability cellular tests. The toxicity of the peptide’s gelators may be correlated to their tendency to self-assemble, but this toxicity disappears once the hydrogel is formed [136]. Actually, peptide-based hydrogels have already shown their potential as a matrix for cell culture experiments creating adequate 3D microenvironments [137,138,139,140]. It has been demonstrated that they promote cell differentiation, stem cell proliferation, support the attachment of different kinds of cells, can be used for angiogenesis assays, and tumor cell migration and invasion tests [141,142,143,144,145,146]. Some hydrogel formulations are already in the market (e.g., Puramatrix, Hydromatrix, TrueGel3D). Last but not least, thanks to their inherent biocompatibility, thixotropic property, and the above-mentioned capability to growth cells, they are promising candidates for applications in regenerative medicine and tissue engineering [139,145,147].

#### 4.1.2. In-Vivo Tests

Once the in-vitro step is validated for the biocompatibility, cell viability and feasibility of the nanopeptides, the in-vivo assays may be performed. First steps consist in performing biodistribution studies to follow the kinetics of uptake, remanence, and clearance within the different organs. They can be performed by functionalizing the peptides with imaging probes such as fluorescent, magnetic or radioactive moieties, or by encapsulating fluorescent drugs within the nanopeptides, for example in the cavitary space within the nanotubes or nanospheres, and to perform in vivo biodistribution kinetic studies by various non-invasive optical, MRI or PET imaging modalities [148]. These studies will be afforded in a rational way in the sections devoted to in vivo targeting and imaging, drug delivery, gene therapy and phototherapy.

### 4.2. Biomedical Applications of Self-Assembled Peptide Nanostructures

**Figure 9:**
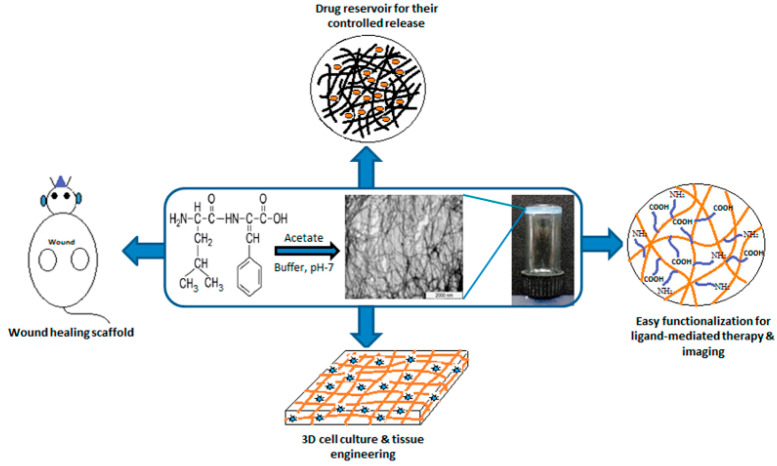
Self-assembling of LΔF dipeptide and their applications in the biomedical field. Adapted with permission from Reference [149].

Self-assembled peptide nanostructures have been successfully applied in a wide range of biomedical applications going from diagnostics till therapy and regenerative medicine purposes. The following paragraphs provide a summary of the main biomedical applications, with a special emphasis to the unique peptide features, including but not limited to: on demand easy production, intrinsic functional groups for further functionalization, high biocompatibility, intrinsic therapeutic potential, cell penetration, adhesion, targeting, proliferation, stimuli-responsive, etc. Indeed, these features make peptide nanostructures promising tools over other synthetic systems for biological applications. It is noteworthy that one unique peptide sequence can give rise to different nanostructures, depending on the peptide concentration and environment conditions, that would be pertinent for different applications as shown in Figure 9. for the case of the dipeptide LΔF, with ΔF a non-protein amino acid, α,β-dehydrophenylalanine [149].

#### 4.2.1. Biosensors

Peptide self-assembled nanostructures are mainly used in the development of electrochemical biosensors aiming at improving the analytical performances (sensibility, selectivity, and limit of detection) by playing two roles: (i) increasing the active surface area of the electrode so as to generate signal amplification or increase the electron transfer (electron mediators); (ii) being functionalized by or encapsulating either the biorecognition element or the signal probes/reporters or both. Thus, they have been employed for direct observation of molecular interactions in a wide range of affinity and enzymatic assays [150]. Castillo-Leon et al. [151] provide an overview of the fabrication and deposition processes of self-assembled peptides onto transducer surfaces, highlighting different functionalization strategies with biorecognition elements (antibodies, enzymes), nanomaterials (gold NP, carbon nanostructures), polymers (poly(3,4-ethylenedioxythiophene) PEDOT, polyaniline PANI) and their application in the biomedical and environmental fields. Some interesting derivatized amino acid and peptide sequences for the construction of electrochemical devices are presented such as Boc-F-OH, H-F-OMe, FF, Cyclo [(Q-L)4], NSGAITIG, and EAK16-II for their ability to self-assemble in controlled nanotubes or nanofibers structures.

Peptide hydrogels have also been successfully employed for the fabrication of biosensors for the encapsulation of bioreceptors (enzymes, aptamers), antigens, cells, and signal reporters (QDs). They create smart bio-interfaces that improve the performances of the diagnostic tool for the determination of a wide variety of compounds going from small molecules of clinical/biological interest (H_2_O_2_, glucose, phenols) till the detection of pathogens, cancer biomarkers, and nucleotide polymorphisms [152,153,154,155]. Both electrochemical and optical biosensors were developed with peptide hydrogels, showing practical advantages such as simple fabrication, efficient diffusion of target molecules, and high encapsulation capacity.

Another interesting advantage in constructing biosensors with self-assembled peptides is their ability to prevent the electrode surface from non-specific adsorption of biomolecules thanks to their inherent antifouling properties [155,156]. As an example, the presence of a self-assembled zwitterionic peptides and IgE-aptamer was demonstrated to provide an ultrasensitive device (LOD 42 fg·mL^−1^) for the IgE determination in biological complex matrix [157].

#### 4.2.2. In-Vivo Imaging

The versatility of nanopeptides allows smart functionalities to be added at desired positions along the peptide chain through well-established chemical syntheses, for their compatibility in biological applications as well as the functionalization of imaging probes to follow their biodistribution and to study targeting as well as monitoring therapy by imaging.

**Figure 10:**
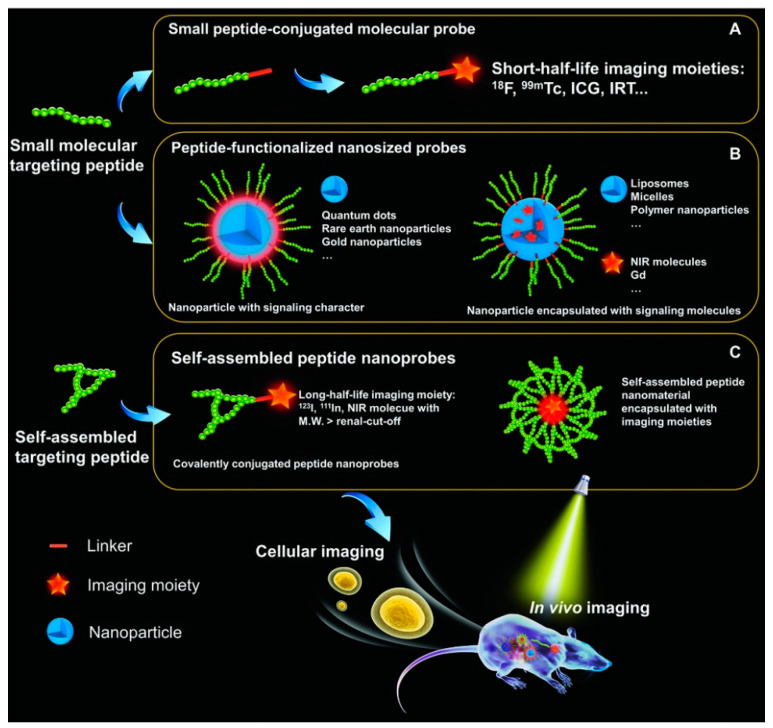
General scheme of targeting peptide-based probes for molecular imaging. (**A**) General conjuga-tion of peptide with a molecular probe. (**B**) Functionalization of nanoparticles with conjugated peptides. (**C**) Self-assembled peptide nanoprobes with additional targeting. Reprinted with per-mission from Ref. [158].

The ability of chemical functionalization of the nanopeptides with imaging probes made them ideal for diagnosis and biodistribution in vivo. The general scheme is summed up in (Figure 10) which covers small peptide imaging probes for optical imaging, newly developed peptides targeted toward important biomarkers, and smart peptide-based nanomaterials [158]. Moreover, Palivan et al. [148]. described an overview of optical, Positron Emission Tomography PET and Magnetic Resonance MR imaging applications using peptidic nanostructures of micelles, nanotubes, nanovesicles, nanofibrils, nanoprobes in vitro and in vivo for the purpose of biodistribution and gene therapy monitoring in a clinical perspective. Finally, peptide-modulated self-assembly is well adapted to tumor nanotheranostics, that integrates diagnosis and therapeutics in a unique platform. The review from the group of Yan [159] illustrates multicomponent cooperative self-assembly for the fabrication of nanotheranostics in vitro and preclinical in vivo and focused on peptide-drugs and peptide-photosensitizers.

##### Positron Emission Tomography (PET) Imaging

Faintuch et al. [160] developed radiolabeled nanopeptides of hybrid oligomers and peptides forming micellar nanospheres, that showed specificity for an animal model of human PC3 prostate cancer cells. Nanobombesin labeling by a pre-targeting system provides an alternative approach for prostate tumor treatment. A pre-targeting system combining streptavidin (SA), biotinylated morpholino (B-MORF), biotinylated peptide Bombesin BBN (B-BBN) with polyethylene glycol (PEG) spacers and a radiolabeled complementary phosphorodiamidate morpholino oligomer (cMORF) for in vivo positron emission tomography (PET) imaging was evaluated in vitro and in vivo.

Nanofibers based on self-assembling peptide analogues were assessed for tumoral uptake and biodistribution in vivo, in real-time, through Positron Emission Tomography Computed X-Ray Tomography (PET/CT) imaging [161]. Peptide precursors were PEGylated to prevent the reticulo endothelial system capture and evolve into an interfibril network for prolonging half-life and favor the tumor retention by Enhanced Permeability Retention EPR. PET/CT imaging employing ^89^Zr-labeled GSH-NFP, showed a supplied tumoral delivery and drug retention, as well as the accumulation of the nanofibers at the tumor periphery as well as in the main organs, for 7 days.

##### Magnetic Resonance Imaging (MRI)

Novel di- and tetra-phenylalanine peptides derivatized with gadolinium complexes assembled in nanofibers have been proposed by Diaferia et al. [162] as potential supramolecular diagnostic agents for applications in MRI. It was observed that in very short FF dipeptide building blocks, the propensity to aggregate decreases significantly after modification with molecular functions such as Gd-complexes. For example, the synthesis, structural and relaxometric behavior of a novel water soluble self-assembled peptide contrast agent based on modified F amino acid by 2-naphthylalanine (2Nal) was developed [163]. The peptide conjugate Gd-DOTA-L6-(2Nal)2 is able to self-assemble in long fibrillary nanostructures in water solution (up to 1.0 mg·mL^−1^). Similarly, fibrillar nanostructures co-assembled in β-sheet from different PAs containing a hydrophobic block and a linker peptide conjugated to 1,4,7,10-tetraazacyclododecane-1,4,7,10-tetraacetic acid (DOTA) were used for improving contrast in MRI [164]. Co-assembly of PAs and polymers into micelles which are more robust than pure peptidic micelles is an effective way to enhance stability, as was shown for gadolinium-decorated poly(ethylene-oxide) PEO pluronic F127 and peptide amphiphile (Pyridil A(pal)-AAAAHHHD) hybrid micelles [165]. The hybrid micelles circulated much longer than small clinical Gd-DTPA molecule. The doxorubicin (DOX) loaded hybrid micelles was also able to suppress tumor growth as therapeutic agent in vivo.

**Figure 11:**
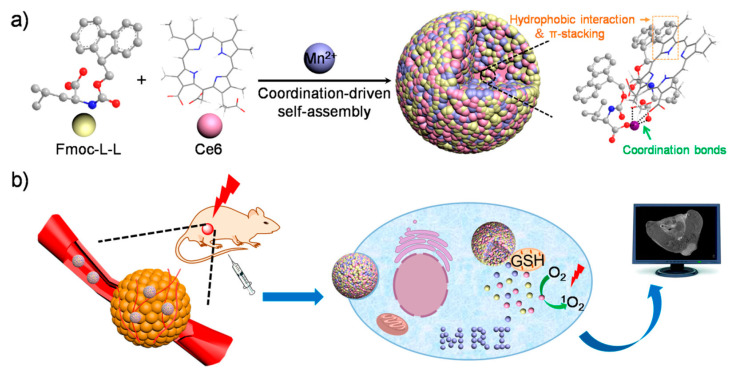
Fmoc L-L coordinated to photosensitizer chlorin e6 nanoplatform for enhanced cellular uptake and tumor accumu-lation in addition to the tumor microenvironment (GSH)-responsive release of drugs for photodynamic therapy PDT and mag-netic resonance imaging agents. (**a**) Synthesis steps of the peptidic nanoplatform with PDT photosensitizer and Mn^2+^ for MRI. (**b**) In vivo PDT with imaging MRI monitoring showing a superior antitumor efficacy and MR and optical imaging capability. A complete tumor eradication after PDT was observed showing their potential as a biocompatible and efficient phototherapy agent. Re-printed with permission from reference [166].

Zhang et al. [166] demonstrated that the nanoplatform of Fmoc L-L coordinated to photosensitizer chlorin e6 was formulated by a one pot mixing 9-fluorenyl methoxy carbonyl-L H (Fmoc-L-L), a photosensitizer (Chlorin e6, Ce6), and Mn^2+^ in aqueous solution to form Fmoc-L-L/Mn^2+^ nanoparticles (FMNPs) obtained from noncovalent interactions (hydrophobic interaction, π-π stacking). The nanoplatform enables enhanced cellular uptake and tumor accumulation in addition to the tumor microenvironment glutathion (GSH)-responsive release of drugs for photodynamic therapy (PDT) and magnetic resonance (MR) imaging agents, resulting in a superior antitumor efficacy and MR and optical imaging capability. The NPs show the beneficial combination of the real-time monitoring of the in vivo delivery and the noninvasive assessment of the therapeutic efficacy (see Figure 11). This integrated platform supplies interesting perspectives as a highly versatile theranostic agent for cancer.

**Figure 12:**
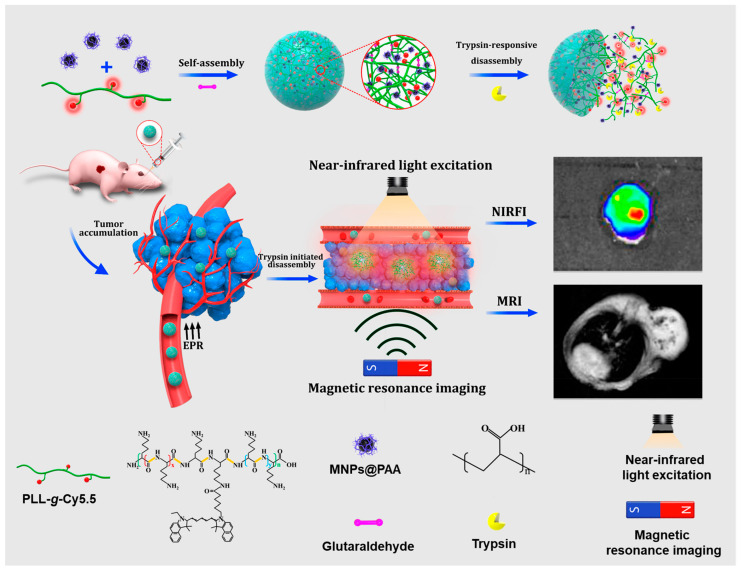
MR imaging and trypsin-responsive substrate/NIRF agents composed of Fe_3_O_4_ magnetic nanoparticles whose surface is decorated with polyacrylic acid and Cy5.5-modified poly-d/l-L (P-d/l-L-g-Cy5.5). Reprinted with permission from Reference [167].

Near-infrared fluorescent (NIRF) and magnetic resonance dual-imaging coacervate nanoprobes were designed for trypsin mapping and targeted payload delivery of malignant tumors by Guo et al. [167] (Figure 12). These nanoprobes are composed of Fe_3_O_4_ magnetic nanoparticles whose surface is decorated with polyacrylic acid and Cy5.5-modified poly-d/l-L (P-d/l-L-g-Cy5.5) leading to MR imaging and trypsin-responsive substrate/NIRF agents, respectively. The poly-d/l-L (P-d/l-L-g-Cy5.5) was self-quenched after construction of the nanoprobes. After hydrolysis of poly-L-L peptide by trypsin inside the tumor cells, the 100 nm nanoprobes were selectively disintegrated into fragmented segments, resulting in a 18-fold amplification of the NIRF intensity compared with the initial nanopeptide as well as strong enhancement of the MR imaging. In vitro and in vivo studies demonstrate that the coacervate nanoprobes display remarkable trypsin-sensitive NIRF and MR dual-imaging capabilities to efficiently map malignant tumors in which trypsin is overexpressed.

##### Fluorescence NIR Imaging

Self-assembled peptides with a various number of phenylalanine (F) amino acid units provide optical properties. A second harmonic generation response is detected in FFF-nanobelts, FF-nanotubes, and FFF-nanospheres [168], with efficient optical frequency conversion from NIR to green and blue light opening the perspective for a new generation of nonlinear optical nanomaterials. Besides, photoluminescent peptide nanotubes can arise from the incorporation of lanthanide complexes during the self-assembly process [169]. The peptide nanotubes and photosensitizer molecules exhibited a high synergistic effect on the enhancement of lanthanide photoluminescence. Moreover, optical imaging can be generated by doping the nanopeptides with fluorescent dyes. For example, a nanostructured hydrogel in which a fluorophore was covalently bound was developed by Li et al. [170] for the controlled release of the anticancer drug taxol, and the in vitro imaging.

**Figure 13:**
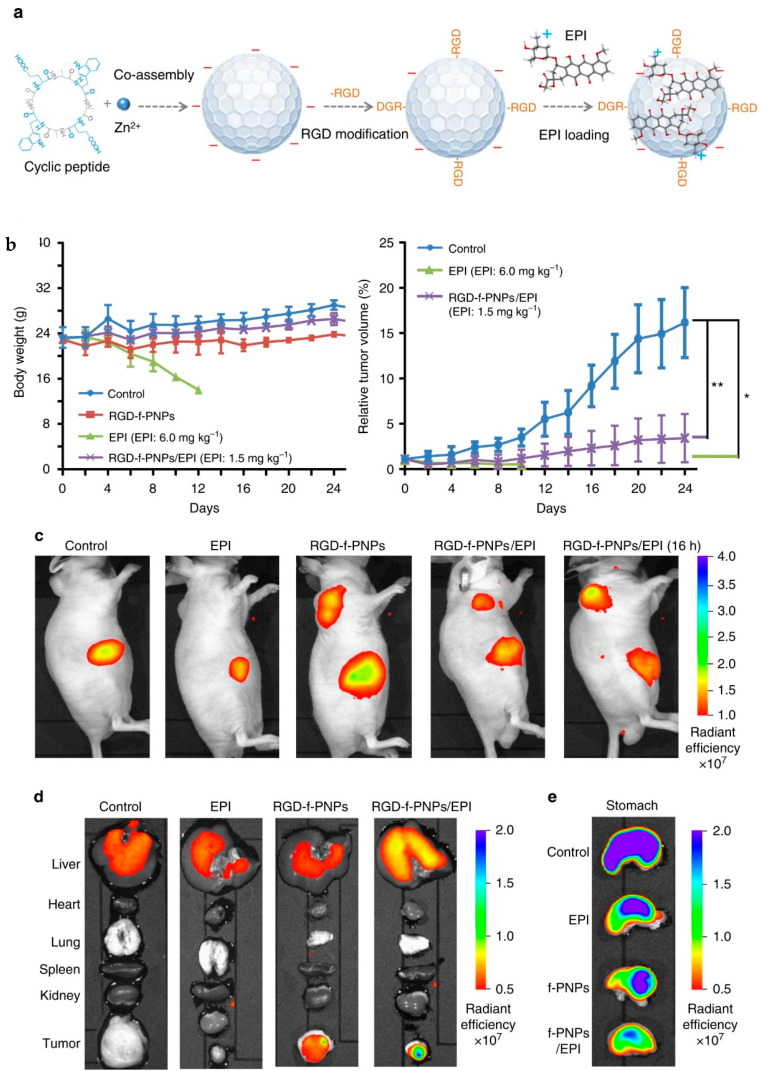
(**a**) Synthesis of RGD-f-PNPs/EPI cyclic octa-peptide cyclo- [(D-A-L-E-D-A-L-W)2-] co-assembled in the presence of Zn^2+^ to form spherical nanoparticles (**b**) Impact in the body weight and the tumor growth after the RGD-f-PNPs/EPI treatment. The results show a significative difference between the control and EPI treatment (* *p* ≤ 0.05) and RGD-f-PNPs/EPI treatment (** *p* ≤ 0.01) (**c**–**e**) Biodistributions observed by in vivo and in vitro NIR fluorescence imaging displayed in addition to the passive liver NP capture, the more specific tumoral uptake from RGD. Reprinted with permission from Reference [171].

Recently, fluorescent self-assembled cyclic peptide nanoparticles (f-PNPs) were developed to form nanospherical structures which combine imaging and drug delivery for esophageal cancer (EC) [171]. Cyclic peptides (D-A-L-E-D-A-L-W) were co-assembled in the presence of Zn^2+^ to form spherical nanoparticles. They were then functionalized with RGD for tumor targeting, and fluorescent drug epirubicin (EPI) for therapy, to generate RGD-f-PNPs/EPI cyclic octa-peptide with cyclo- [(D-A-L-E-D-A-L-W)2-]. They were proved to supply quantum confinement of the structure to become fluorescent in the visible and NIR window, and to allow the monitoring of the drug delivery to tumor sites and of the therapeutic responses. In vitro and histology tests proved significantly reduced cardiotoxicity and improved anti-tumor activity compared to the drug alone. This unique nanoparticle system may lead to potential approaches for bioorganic fluorescence-based delivering, imaging, and drug release tracking (Figure 13).

As demonstrated with previous multimodal theranostic peptide based nanoplatforms presented in this review, multimodal association of multiple imaging probes, targeting moieties and therapeutic substances should provide efficient therapeutical strategy with clinical envision. These peptidic multicomponent synergetic self-assembly is a promising approach for nanotheranostic strategy for innovative therapy.

#### 4.2.3. Drug Delivery

To overcome the side effects induced by chemotherapies, to improve the solubility, bioavailability and uptake of drugs, to facilitate the barrier transfer, among others, great progress is being made in the research of drug carriers. In this context nanocarriers have gained much attention in the last decades since they can circumvent some problems encountered by conventional drug delivery systems associated to non-specificity, burst release or damage of normal cells [172]. They should improve the pharmacokinetics, bioavailability and therapeutic efficacy of the drug while providing preferential accumulation in the targeted tissue so as to decrease the side effects. Since one can modulate their physicochemical properties and thus their behavior, the main parameters to consider in designing nanocarriers are their composition (either organic, inorganic, or hybrid), their size and surface charge density, their geometric shape and curvature, and their ability to be stimuli responsive (pH, temperature, enzyme, light, etc.) so as to release the drug in a precise environment or conditions [172,173].

Peptides self-assembly into nanocarriers offers many advantages for drug delivery, such as the ability to carry both hydrophilic and hydrophobic drugs, with a high efficiency of drug loading and a low ratio of drug loss, while having the capacity for sustained drug delivery at the targeted site, and high biocompatibility and stability. In addition, nanopeptides can be customized to incorporate peptide sequences or targeting moieties for specific cell targeting and responses making them attractive for drug-delivery applications. Thus, several structures of peptide assemblies have been recently described as drug delivery systems in different reviews [174,175,176]. The work of Pentlavalli et al. [174] was devoted to the study of peptide self-assembly mechanisms and the influence of external stimuli (pH, ionic strength, temperature, enzymes) inducing the delivery of the drugs. Tesauro et al. [175] focused on amphiphilic peptides design, emphasizing the wide range of sequence selection to provide the peptides with features such as specific targeting, cleavage, or stimuli responsivity enhancing the uptake, specific release of the drug and thus therapeutic efficiency. Gupta et al. [176] highlighted the potential of single amino acid and ultrashort peptides (di- and tri- peptides) and their derivates as drug/gene carriers.

Currently the most common nanostructural peptide arrangements used as nanocarriers for drug delivery are nanoparticles, nanoformulations [177], micelles [178], vesicles [179,180], nanotubes [134,181,182] and hydrogels [152,183,184]. They have been proved to successfully perform the carry and controlled release of both hydrophobic and hydrophilic drugs including anti-cancer, anti-fungal, anti-infectious and antibiotics as for example doxorubicin, curcumin, paclitaxel, cisplatin, mitoxantrone, streptomycin. In Table 1 are collected specific sequences that introduce special features to the peptides for drug delivery and biological applications. Note that these sequences can form part of a larger peptide sequence and be as well further derivatized with other molecules like polymers (PEG), phospholipids, cholesterol, contrast imaging agents (DOTA).

Cyclic peptides LyP-1 (H-C1-C2-G3-N4-K5-R6-T7-R8-G9-C10-OH) conjugated to PEG–PLGA nanoparticles (LyP-1-NPs) to create a hybrid system were synthetized for targeting drug delivery and applied to lymphatic metastatic tumors [199]. In vitro, cellular uptake of LyP-1-NPs enhanced four times, and in vivo the uptake of LyP-1-NPs in metastasis lymph nodes was about eight times compared to non-targeted NPs.

Cheng et al. synthetized and evaluated a responsive therapeutic peptide, consisting of a peptide substrate of a metalloproteinase-2 (MMP-2) matrix, and a tumor targeting anti-PDL1 DPPA-1 peptide, which co-assembled in a micellar structure and encapsulated an inhibitor of dioxygenase, for in vivo dual-targeted cancer immunotherapy [179]. The hybrid nanostructure was activable through the weakly acidic tumor environment, and further collapsed due to the cleavage of the peptide substrate that was highly expressed in tumor stroma. The localized release of DPPA-1 and antitumor inhibitor favored T lymphocytes activation, leading to the slowdown of melanoma growth and increase of overall survival. This study evidences the interest of dual-targeted cancer immunotherapy through functional peptide assembling nanoparticles with designed features.

Another important aspect that has recently emerged in the area of anticancer drug delivery is the use of peptides as active drugs for therapeutics [174]. Here the concept is to design a peptide sequence which self-assembles due to environmental factors once it is into the tumoral cellular compartment and the formed nanostructure results in a cellular disruption causing the cellular death. This self-assembly can be induced by a pH, ionic strength, or temperature change or by using an enzymatic triggered self-assembly process thanks to the presence in the peptide sequence of a cleavable part which is a substrate of the specific enzyme of tumoral cells [195,200,201,202]. This phenomenon of in-cell self-assembling starts to be known as “peptide reverse self-assembling” [203].

Finally self-assembled peptide nanostructures have not only been used as drug delivery systems for the treatment of cancer but also in other physio-pathological environments including central nervous, cardiovascular, intra-ocular, bone, wound healing systems, and in the fight against Human Immunodeficiency virus (HIV) as recently reviewed in the works of Eskandari [183] and Pentlavalli [174].

#### 4.2.4. Gene Therapy

An efficient gene delivery system should have the general following characteristics: (1) protection of the DNA content thanks to nanovectors, (2) stability after administration to the action site, (3) access and penetration into the target cell/tissue, and (4) after internalization, release of the nucleic acid within the action site [188]. For classical non-viral vectors, the main difficulty is their bad in vitro to in vivo translation and their toxicity. Peptide-based vectors enable to overcome delivery barriers, including the host’s immune response, and to reduce cytotoxicity [158]. Recent works describing the gene therapy potential of nanopeptides have been extensively reviewed by the group of Palivan et al. [148] and interesting gene cargos made of ultrashort peptides are presented in the report of Gupta et al. [176].

Peptide-based nanostructures can be co-assembled with nucleic acids and with imaging probes, for the delivery of nucleic acids, to host cells, or improve the specificity and sensitivity of probes in diagnostic imaging. In particular, these nanopeptides could be micelles with DNA encapsulation, as well as bilayers vesicles, nanofibers, nanotubes; these nanoparticles of peptiplex (nucleic acid combined with peptides) enable efficient internal DNA encapsulation and transfection in vitro [148].

To optimize gene therapy, cell penetrating peptides (CPP) are often functionalized and applied against hypoxic–ischemic brain injury, spinal cord tumors, and in ischemic heart and lung diseases. Pure peptidic nanoassemblies studied in vivo are peptiplexes which are often covalently functionalized with poly(ethyleneglycol) (PEGlyated) to increase their nanosize, reduce protein corona and improve their in vivo stealthiness with prolonged half-life and with stability [189,190,191]. Biodegradable poly-L-L (PLL) derivatives were one of the first cationic cell penetrating polypeptides. PEGylated PLL/siRNA peptiplexes were applied as anti-angiogenesis gene therapy in hepatocellular carcinoma and showed high anti-tumor efficacy [192]. Moreover, one of the most versatile and promising in vivo gene transfer vectors with cell penetrating properties are peptiplexes based on various lengths of oligoarginine described in the review of Midoux et al. [193].

Notably, peptide dendrimers, highly branched and star-shaped macromolecules with great molecular uniformity and monodispersity, are well-studied examples for in vivo non-viral gene delivery, based on their cell penetrating properties, as well as their potential to facilitate intracellular delivery of the genetic payload as described by Kesharwani et al. [204]. Furthermore, elastin-like polypeptides (ELPs) with various favorable properties such as water solubility, biocompatibility, non-toxicity, together with reversible temperature phase transition were widely investigated for gene delivery over the past decade by Smits et al. [205]. Cyclic octa and di-peptides nanotubes (cPNT) were also developed for gene delivery. For example, Hsieh et al. in 2014 [196] designed and assessed in vitro to in vivo the Cyclo-(D-W-Y) cPNTs, of 100–800 nm widths and 1–20 μm lengths, so as to successfully deliver plasmid DNA (using luminescent reporter genes) on mice with an efficient biodistribution and transfection by oral delivery.

#### 4.2.5. Phototherapy

**Figure 14:**
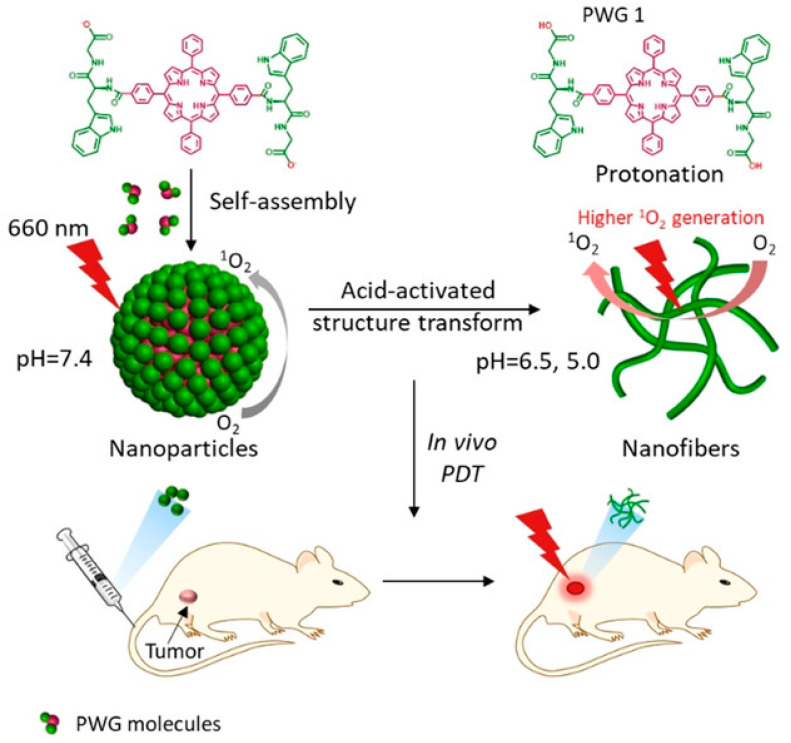
A schematic representation of the self-assembly and fibrillar transformation of the acid-activated peptide-porphyrin (PWG) nanoparticles and their application in PDT. The structure transformation in acidic medium enables the accessibility of the laser irradiation to the porphyrin, inducing the singlet oxygen generation in a specific way. Reprinted with permission from Reference [206].

For phototherapy, the peptide-based nanostructures are usually decorated with compounds acting as photosensitizers and creating the so-called hybrid system. The photosensitizer is either an organic molecule (porphyrin) or another nanomaterial (metal nanoparticles). The role of the peptide-based nanostructure is to facilitate the transport, targeting and uptake of the photosensitizer and to increase their retention and accumulation in the target tissue and thus, increase the anti-tumor efficiency. This hybrid system can take advantage of the stimuli-responsive (pH, redox, enzymatic) capabilities of self-assembled peptides, which induces structural transformations enhancing the efficacy of the phototherapy. This strategy was adopted by Sun et al. [206] in the development of an acid-activable peptide-porphyrin hybrid system for photodynamic therapy (Figure 14). The hybrid system presented a nanoparticular structure under physiological conditions which turned into nanofibers under acidic environments, i.e., tumor microenvironment and lysosomes. The pH-responsive transformation into fibrillar structure exposed the porphyrin, which was at the core of the nanoparticle at physiological pH, allowing its laser irradiation and inducing the controlled and enhanced generation of singlet oxygen for the photodynamic therapy without off-target side effects.

Zou et al. [207] has also formulated an interesting self-assembled peptide-porphyrin (TPP-G-FF) to form nanodots with a porphyrin photosensitizer delivery for photothermal therapy (PTT). In vivo biodistribution studies and thermal and photoacoustic in vivo imaging were studied showing a major expected liver as well as 10% tumoral uptake. These nanodots are particularly soluble in aqueous media, with a stable scaffold against dilution and irradiation, and high light-to-heat conversions (54.2%) leading to photoacoustic and thermal imaging features. They have been proved to prevent the growth of tumor in a preclinical mice model.

#### 4.2.6. Nanopeptides as Drugs: Antiviral and Antibacterial Effects, and Vaccine Engineering

Self-assembled peptide nanostructures (β-sheets, α-helices, peptide amphiphiles, amino acid pairing, elastin like polypeptides, cyclic peptides, short peptides, Fmoc-protected peptides, and peptide hydrogels) are well adapted for further application in vaccine engineering as developed in the review of the group of Eskandari [183]. Cyclic di-, tri-, tetra-, hexa-, octa-, and decapeptides with various amino acid sequences, enantiomers, and functionalized side chains can be also applied for antiviral and antibacterial drugs as detailed in the work of Hsieh et al. [182].

Some works evidenced peptide nanostructure’s antibacterial activity. Gao et al. [208] developed amphiphiles self-assembled nanorods of peptides against bacterial activity for efficiently treating antibiotic-resistant bacteria in vitro. A soluble antibiotic (ciprofloxacin) and a hydrophobic tripeptide ((D)L-F-F) self-assembled into supramolecular nanostructures to form a macroscopic hydrogel [209] exhibiting a mild anti-bacterial activity against Gram-negative bacteria and importantly no major haemolytic toxicity on human red blood cells or in mouse fibroblast cell cultures. Antibiotic effect of cyclic PNT was demonstrated by Ghadiri group in 2001 [210]. The PNTs was demonstrated efficient for surface coating to prevent microbial colonization inn health care.

Antibacterial activity of self-assembled diphenylalanine emerges as the minimal supramolecular assembly [211]. The diphenylalanine nanoassemblies completely inhibit bacterial growth, induce disruption of bacterial morphology, and cause membrane permeation and depolarization. Indeed, Schnaider et al. [212] evidenced the specificity of nanopeptides to interact with microbial membranes and the development of antibacterial materials (e.g., ultrashort cationic hybrid naphthalene derived peptides) and these study lead to the development of antimicrobial agents and materials. For example, ultrashort non-steroidal inflammatory drug peptides attached to diphenyl lysine (FFKK-OH) peptide self-assembled in hydrogel nanosponges [213] or self-assembly of cationic multidomain peptide hydrogels [214] have been able to destroy in vitro bacterial cultures.

Concerning vaccine strategy, introduction of autophagy inducing transactivator of transcription TAT autophagy inducing peptides eliminated latent HIV infection in vitro in selective latently HIV-infected CD4+ T cells (major reservoir of HIV latent infection) via lipid-coated hybrid polylactic co-glycolic acid (PLGA) nanoparticles for a strategy to prevent the reactivation of the virus and new infection in bystander cells in a vaccine type manner [215].

#### 4.2.7. Tissue Engineering and Regenerative Medicine

Tissue engineering is based on the growth of cells into a scaffold that has the mechanical and biological features well-adapted to this end, close to those of extracellular matrix (ECM). Thus, growing within the scaffold matrix, tissue cells are regenerated or prepared to replace skin, bone, cartilage, or part of an organ. Tissue engineering aims at developing materials and methods to promote cell differentiation and proliferation toward the formation of a new tissue [181]. Among the different materials proposed to be scaffolds, peptides are one of the most promising biomaterials for tissue engineering and regenerative medicine since the main signaling language in the ECM is mediated via peptide epitopes and, as mentioned previously, they possess the thixotropic property enabling an easy way of administration directly into the injured location [147,174]. Indeed, thanks to these features, along with its similarity to ECM that limits chronic inflammation responses or immunological reactions and toxicity, peptides are postulated as improved scaffolds compared to polymers and biopolymers that have been used as artificial scaffolds in this domain [216]. In addition, self-assembled nanostructures are well suited for regenerative issues thanks to their structures and mechanical properties which enable them to fulfill the main challenges of tissue engineering: (1) adaptable mechanical strength suitable for different parts of the body, (2) porosity for lighter weight and ensuring sufficient blood and oxygen supplies and waste transport, and (3) biocompatibility to prevent immune rejection and inflammation [176,181]. Thus, for example nanotube and nanofiber structures from peptide sequences such as C_16_A_4_G_3_LRKKLGKA; KLD12; and V_3_A_3_E_3_ serve as guidance for the growth of vessels, cartilages, or bones [217,218,219]. The hydrogel state of self-assembled peptides is also performant in tissue modeling in diverse medicine areas such as oncology [145] or cardiovascular [143]; as well as tissue engineering and regeneration [147]. For example, the 3D nanofiber peptide hydrogel formed by RADA16-I peptide is similar to the structure naturally present in the ECM, and possesses features of cell attachment, proliferation, and differentiation, proved to be efficient for bone regeneration showing an immediate hemostasis and accelerative osteosis [220]. This peptide RADA16 therefore seems to play a pivotal role in regenerative medicine since it has also proved its value for liver, cardiovascular and neuronal tissue regeneration being alone or in combination with other peptide sequences and molecules [202,220,221].

## 5. Conclusions

In this review, we have highlighted from the very rich literature the growing importance of peptide nanostructures for biomedical applications, as well as the need for powerful synthesis processes and in-vitro and in-vivo physico-chemical and biological characterization methods.

We described first the impact of developing new synthesis methodologies, such as flow chemistry, for the efficient production of peptide sequences in high purity, good yields and short time, leading to their convenient functionalization with (bio)molecules. Furthermore microfluidic appears as a real tool for monitoring the self-assembly of peptides into well-designed nanostructures, through kinetics and thermodynamics control. Thanks to milli- and micro-flow systems, the literature allows to envisage in a near future to couple synthesis, functionalization and self-assembly in a unique flow device, allowing for a total control of the nanoarchitecture.

As the peptide self–assembly is dictated by physical driving forces and environmental factors, the design of the peptide sequences is a main parameter to generate the expected nanostructure. As evidenced with short peptide families, a deep characterization in terms of global peptide structure and their self-assembly into nanoarchitectures is therefore crucial. The combination of various classical microscopic, spectroscopic and scattering methods, along with computational and theoretical approaches, provides a precise physico-chemical characterization as well as self-assembling mechanism elucidation. The emergence of separation methods coupled to efficient detection modes highlights their interest to propose in one single experiment various information, going from deep characterization of the peptide sequence, identification and control of the self-assembly process as well as possible interactions in a medium mimicking biological conditions (such as protein corona). Then the in-vitro and in-vivo biological characterizations of the peptide nanostructures complement the overall information to help for the determination of their biocompatibility, toxicological effects and main biological activity and, consequently, for the selection of efficient nanostructures for biomedical applications.

This review finally presents the principal biomedical applications of self-assembled peptide nanostructures, which evidences the high potential of such architectures in this domain, going from biosensors, in-vivo imaging, drug delivery, gene- or photo-therapy, antiviral and antibacterial therapies, to vaccine and tissue engineering and regenerative medicine.

Therefore, this review demonstrates the need for interdisciplinarity and development of emerging methodologies in terms of synthesis and characterization so as to help for the rapid and efficient design of peptide nanostructures, which will open the way for a very broad field of applications.

## Figures and Tables

**Table 1:** Some examples of specific peptide sequences that introduce special features for drug delivery and biological applications.

Special Feature	Example of Peptide Sequence	References
***Cell targeting*** (recognition of membrane receptors)		
α,β-Integrins (overexpressed in endothelial cells of the tumor; ovarian, breast, colon, melanoma cancers)	RGD	[173,175]
Somatostatin (overexpressed in neuroendocrine tumors)	OCT (Octreotide) TATE (Octreotate) KE108	[175]
Bombesin (overexpressed in prostate, breast, small cell lung, ovarian, and gastrointestinal cancers)	[7-4]BN	[162,175]
Cholecystokinin (overexpressed in neuroendrocrine tumors, and gastroenteropancreatic tumors)	CCK8	[175]
***Cell penetration*** (facilitates the internalization and thus enhancing the uptake of the drug)	Cell penetrating peptides (CPPs) are usually cationic, mainly composed of basic amino acids. More than 1700 sequences were validated. Peptides derived from α-helical domain of the Tat protein as for example GRKKRRQRRRPQ (TAT dodecapeptide) Peptiplexes Peptide dendrimers	[185,186,187,188,189,190,191,192,193]
***Sustained release***	C16V_2_A_2_E_2_ (release kinetics over 40 h) C16V_3_A_3_K_3_K(folate)-NH2 (release over 5 days) Palmitoyl-A_4_G_3_E_3_ (release over 1 week) M-ΔF	[183]
***Stimuli-responsive*** (the stimuli provoke a structural reorganization of the peptide nanostructure facilitating the controlled release of the drug)		
pH	-THAGYLLGHINLHHLAHL(Aib)HHIL (TH peptide; neutral at physiological pH, become positively charged in acidic medium as in tumor cells) -Oligoarginine (highly charged segment) + β-sheet (self-assembled segment)	[179,194,195,196]
Enzymes	-AAN (specific sequence substrate for Legumain endoprotease which enhances the drug uptake in the targeted tumor) -C_10_H_7_CH_2_C(O)-phe-phe-*NHCH*_2_*CH*_2_*OH* (the cleavage of the *butyric diacid* induces the hydrogelation of the FF inside the cell causing the cell death) -C16-V_3_A_3_E_3_ sustained release activated by enzyme degradation of the peptide nanofiber	[108,171,179,197,198]
Light	After functionalization of the nanopetide with a photosensitizer	[166,196]
